# Platelet-derived TLT-1 promotes tumor progression by suppressing CD8^+^ T cells

**DOI:** 10.1084/jem.20212218

**Published:** 2022-10-28

**Authors:** Tarun Tyagi, Kanika Jain, Timur O. Yarovinsky, Michael Chiorazzi, Jing Du, Cecilia Castro, Jules Griffin, Asawari Korde, Kathleen A. Martin, Shervin S. Takyar, Richard A. Flavell, Abhijit A. Patel, John Hwa

**Affiliations:** 1 Yale Cardiovascular Research Center, Department of Internal Medicine, Yale School of Medicine, New Haven, CT; 2 Department of Immunobiology, Howard Hughes Medical Institute, Yale School of Medicine, New Haven, CT; 3 Department of Biochemistry, Cambridge University, Cambridge, UK; 4 Pulmonary Critical Care, Yale Internal Medicine, New Haven, CT; 5 Yale Therapeutic Radiology, Yale Cancer Center, New Haven, CT; 6 Yale Cancer Center, New Haven, CT

## Abstract

Current understanding of tumor immunosuppressive mechanisms forms the basis for modern day immunotherapies. Immunoregulatory role of platelets in cancer remains largely elusive. Platelets from non-small cell lung cancer (NSCLC) patients revealed a distinct activation phenotype. TREM-like transcript 1 (TLT-1), a platelet protein, was increased along with enhanced extracellular release from NSCLC platelets. The increased platelet TLT-1 was also evident in humanized mice with patient-derived tumors. In immunocompetent mice with syngeneic tumors, TLT-1 binding to T cells, in vivo, led to suppression of CD8 T cells, promoting tumor growth. We identified direct interaction between TLT-1 and CD3ε on T cells, implicating the NF-κB pathway in CD8 T cell suppression. Anti–TLT-1 antibody rescued patients’ T cells from platelet-induced suppression ex vivo and reduced tumors in mice in vivo. Clinically, higher TLT-1 correlated with reduced survival of NSCLC patients. Our findings thus identify TLT-1 as a platelet-derived immunosuppressor that suppresses CD8 T cells and demonstrate its therapeutic and prognostic significance in cancer.

## Teaser

Platelets from NSCLC patients express and release high amounts of TLT-1, a platelet-specific TREM family member, that promotes tumor growth by suppressing CD8 T cells. Antibody-mediated blockade of platelet TLT-1 reduces tumor progression in mouse and rescues NSCLC patients’ CD8 T cells ex vivo.

## Graphical Abstract

**Figure:**
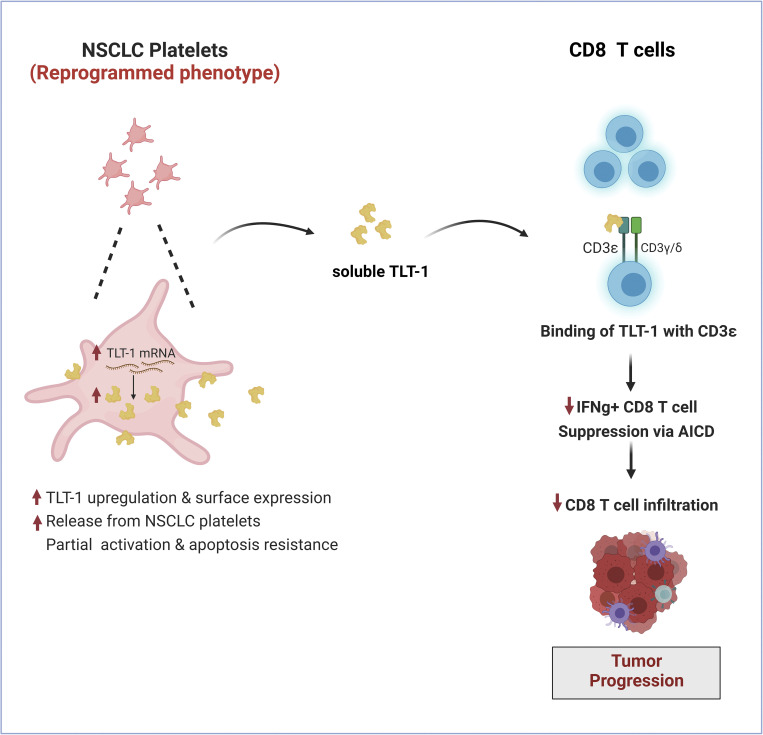

## Introduction

Tumors utilize immunosuppressive mechanisms to restrict effective immune response, facilitating cancer progression and metastasis (Galon and Bruni, 2020; Kim et al., 2006; Motz and Coukos, 2013), and there is growing interest in unraveling this complexity (Abdul Pari et al., 2021; McAllister and Weinberg, 2014). Targeting tumor-associated immunosuppression has shown clinical benefits against several solid tumor malignancies, including non-small cell lung cancer (NSCLC), renal carcinoma, melanoma, bladder cancer, and urothelial cancer (Chen and Mellman, 2013; Egen et al., 2020; Grosser et al., 2019; Mahoney et al., 2015). While most studies on tumor-associated immunosuppression have focused mainly on regulatory T cells, myeloid-derived suppressor cells, and dendritic cells, one of the most abundant circulating cells, i.e., platelets, has remained largely unexplored.

Once thought to have a role only in thrombosis, platelets are increasingly being reported to affect diverse pathophysiological processes including immune dysfunction (Garraud and Cognasse, 2015; Maouia et al., 2020; Semple and Kapur, 2020; von Hundelshausen and Weber, 2007). Elevated platelet counts are associated with several malignancies (Bailey et al., 2017; Ikeda et al., 2002; Pedersen and Milman, 1996; Stone et al., 2012; Tranum and Haut, 1974; Wang et al., 2018), and many studies support a role for platelets in tumor metastasis (Borsig et al., 2001; Coupland et al., 2012; Haemmerle et al., 2018; Haemmerle et al., 2017; Kim et al., 1998; Palumbo et al., 2005; Yu et al., 2014). Previous studies suggest crosstalk between tumor cells and platelets including tumor cell–induced platelet activation in vitro (Goubran et al., 2014; Haemmerle et al., 2018; Heinmoller et al., 1996; Pather et al., 2019; Xu et al., 2018). However, a comparative study between benign and malignant ovarian tumors found that platelets are not hyper-reactive in cancer patients (Feng et al., 2016). Moreover, circulating platelets were found to be “educated” by tumor in NSCLC cancer patients in whom the platelet transcriptome profiles were substantially altered (Best et al., 2015; Best et al., 2018). However, few studies have explored the effect of tumor on platelet morphology and phenotype in cancer patients. The abundance of platelets in circulation and the ability of platelets to release bioactive factors to regulate other cell types warrants investigation into possible roles in antitumor immunity. Moreover, platelet to lymphocyte ratio has been reported to correlate with clinical outcomes in some solid tumor patients (Ma et al., 2018; Zhang et al., 2018).

Platelets express a relatively limited pool of proteins, which includes those involved in immune responses and potential immunomodulators including triggering receptor expressed on myeloid cell–like (TREM-like) transcript-1 (TLT-1; Balkwill, 2004; Ferrer-Acosta et al., 2014; Garraud and Cognasse, 2015). TLT-1 is expressed exclusively by platelets and megakaryocytes (Schmoker et al., 2020; Washington et al., 2009). TLT-1 is involved in platelet activation responses (Washington et al., 2009), can be released from activated platelets (Smith et al., 2018), and plays an important role in hemostasis and sepsis (Branfield and Washington, 2021; Derive et al., 2012). Importantly, TLT-1 bears an immunoglobulin V type (IgV) domain (Branfield and Washington, 2021), which is a common feature of soluble immunocheckpoint ligands/receptors (Basak et al., 2020; Collins et al., 2005). Although platelets only carry a small pool of mRNAs, TLT-1 is reported to be one of the most abundant transcripts (Senis et al., 2007). Recently, in a large-scale computational study on previous tumor datasets reporting markers of T cell exhaustion, TLT-1 (*TREML1*) was among the top upregulated markers associated with T cell dysfunction (Jiang et al., 2018); however, its role in antitumor immunity has not yet been explored.

We set out to elucidate the platelet phenotype in NSCLC patients, with a focus on platelet TLT-1. We investigated its potential immunoregulatory role in cancer using a combination of in vivo and in vitro studies. We identified TLT-1 as a novel platelet-derived immunosuppressor in NSCLC with potential therapeutic and prognostic significance.

## Results

### Human NSCLC platelets express and release elevated TLT-1 and display a distinct phenotype

**Figure S1.**
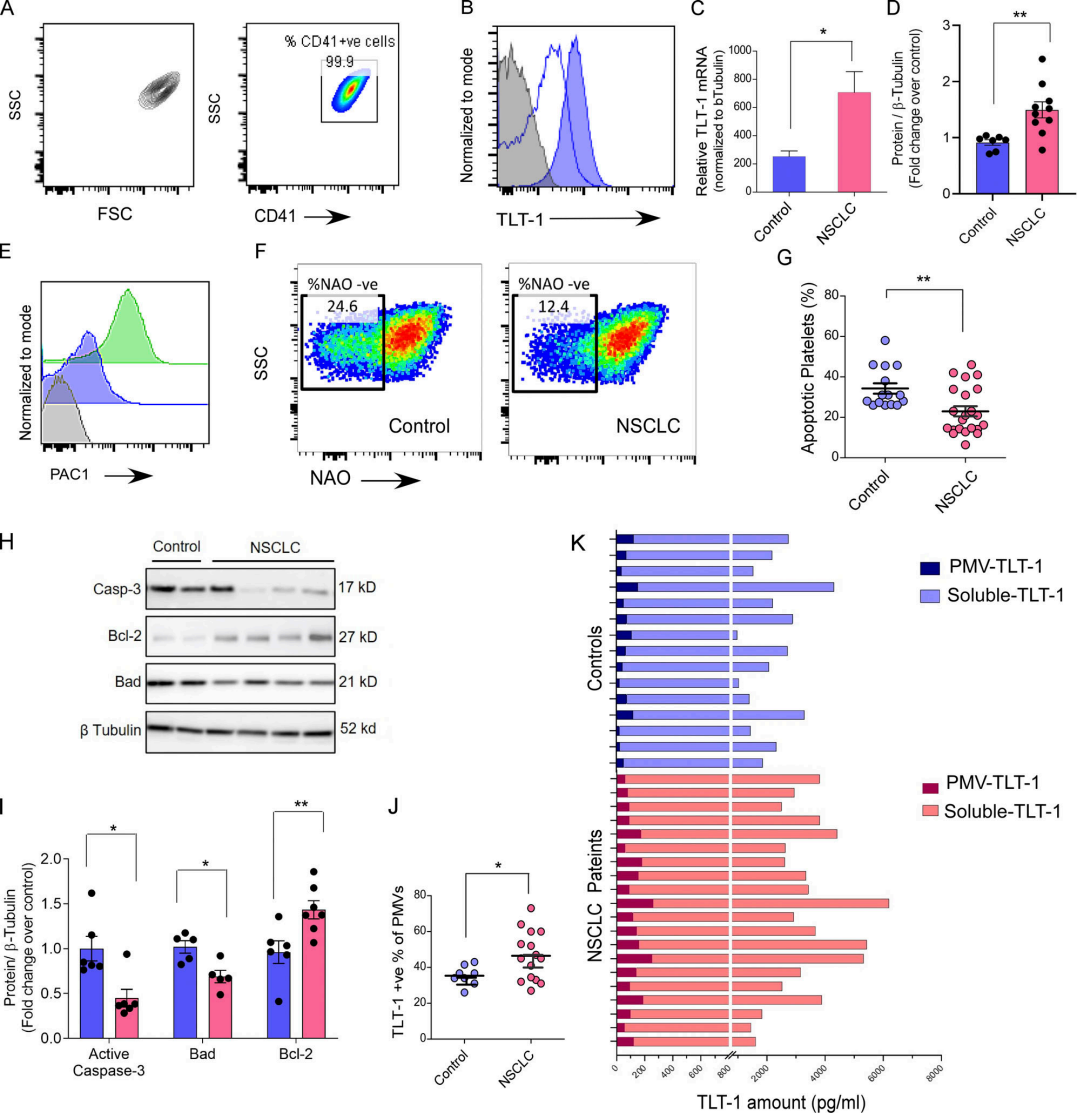
**The platelet purity, gating strategy, and human NSCLC platelet assays.** **(A)** Representative contour or pseudocolor plots for gating strategy (left) and platelet purity (CD41^+^) are shown for FC assays performed on washed platelets. **(B)** The FC histogram overlay showing washed platelets stained with anti–TLT-1 (solid blue) or its isotype control (unfilled blue) and the unstained control sample in solid gray. **(C)** The quantitation (mean ± SEM) of TLT-1–relative mRNA normalized to β-tubulin in individual samples. Statistical significance tested by Mann–Whitney test. **(D)** Quantitation of total TLT-1 platelet expression by Western blot. Statistical significance tested by Mann–Whitney test. **(E)** The FC histograms overlay for positive control for PAC1 marker where washed healthy platelets were stained for PAC1 after activation with ADP (green) or without any external stimulation (blue). The signal from unstained platelets is shown in gray. The events (30,000) were acquired at low flow rate and data analyzed by FlowJo. **(F and G)** Representative FC pseudocolor plots of washed platelets from NSCLC patients and controls show the NAO-stained platelets where NAO-ve population represents ex vivo apoptotic platelets with their quantitation (G; *n* = 15 for control and *n* = 24 for NSCLC). The assay measures the loss of mitochondrial integrity by measuring NAO fluorescence loss from platelets. Statistical significance tested by Mann–Whitney test. **(H and I)** Representative Western blot–based analysis (H) of total platelet expression of activated Caspase 3, Bax, and Bcl2 proteins in NSCLC and control and their quantitation (I) normalized to loading control. The band densities were measured by ImageJ software and normalized to β-tubulin (loading control). **(J)** Quantitation of PMV expressing TLT-1 as measured by FC. Statistical significance tested by Mann–Whitney test. **(K)** ELISA-based comparison of quantitation of TLT-1 in isolated microvesicle fraction versus soluble fraction representing microvesicle-depleted plasma. Each bar represents one patient or control sample as indicated. The data in this figure are presented as FC histograms or as vertical bars with either individual values (in K), or with mean ± SEM. Significant differences are indicated with asterisks (*, P < 0.05; **, P < 0.01; ***, P < 0.001) and correspond to two-tailed non-parametric Mann–Whitney test. The data represent a minimum of three independent experiments. Source data are available for this figure: SourceData FS1.

**Figure 1.**
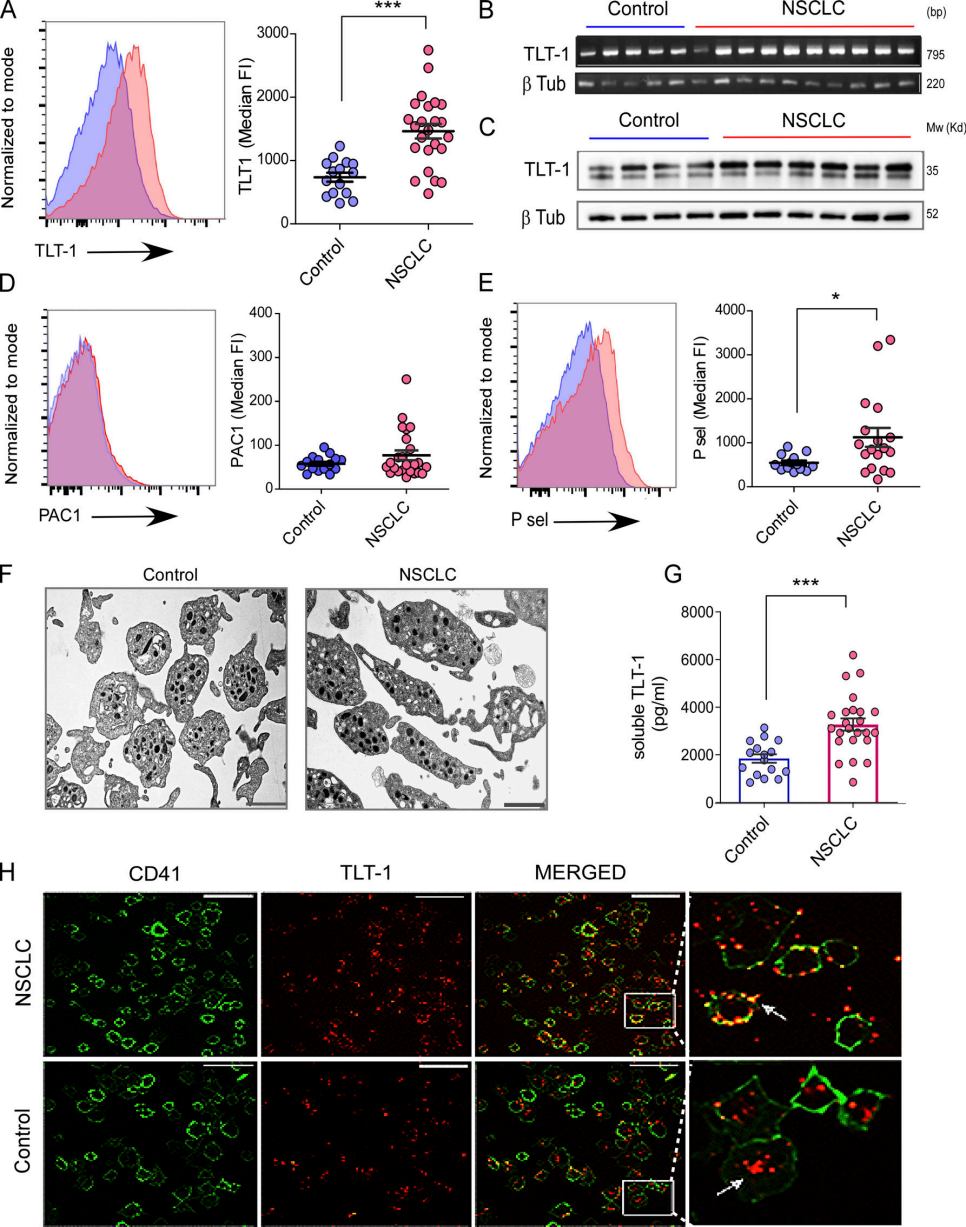
**Human NSCLC platelets express and release elevated TLT-1 and display a distinct phenotype. (A)** Representative FC histograms of isolated platelets from NSCLC patients and controls for surface TLT-1 (*n* = 24 for NSCLC, *n* = 15 for control), with their respective quantitation of median fluorescence intensities (Median FI) in the right panel (statistical significance tested by Mann–Whitney test). **(B)** Platelet TLT-1 mRNA levels in controls and NSCLC patients shown as the respective RT-PCR bands on agarose gel (upper panel); the band size is indicated by bp. **(C)** The immunoblot images show comparison of TLT-1 total protein expression in platelets from controls or NSCLC patients on Western blot, with β-tubulin (β-tub) as loading control as shown. **(D)** Representative FC histograms (left) of isolated platelets from NSCLC patients and controls for surface activation receptor (α_IIb_-β_3_), and respective quantitation (right) measured by PAC1 assay (*n* = 24 for NSCLC, *n* = 15 for control). Statistical significance tested by Mann–Whitney test. **(E)** P-sel surface expression with representative FC pseudocolor plots (left) and quantitation (right) of washed platelets from NSCLC patients and controls (*n* = 15 for control and *n* = 24 for NSCLC). Statistical significance tested by Mann–Whitney test. **(F)** Representative transmission EM micrographs for control and NSCLC platelets (*n* = 4 in each group) shown (scale bar, 1 µm) from samples from each group. The washed platelets were fixed and negatively stained as described in methods. **(G)** Quantitation soluble TLT-1 in plasma in control (blue) and NSCLC groups (statistical significance tested by Mann–Whitney test). **(H)** Activated and adhered platelets from NSCLC or control groups as observed by confocal microscopy showing TLT-1 being released by NSCLC platelets. Representative micrographs are shown with zoomed in portion of merged image on the extreme right. The scale bar represents 15 µm. The data are presented as scatter plots indicating mean ± SEM. The P values shown correspond to non-parametric Mann–Whitney test. All datasets represent a minimum of three independent experiments. Significant differences are indicated with asterisks (*, P < 0.05; ***, P < 0.001). Source data are available for this figure: SourceData F1.

Patients with advanced NSCLC (Table S1) were prospectively recruited (*n* = 42), and their freshly isolated platelets were studied. Platelet counts (249 ± 85 × 10^9^/liter) in the patients were within the normal range (150–450 × 10^9^/liter). Platelets were assessed for TLT-1 expression, activation status, morphology, and apoptosis. We evaluated the surface expression of TLT-1 and other activation markers on freshly isolated platelets from patients (NSCLC) and a control group of age-matched healthy volunteers (control). Platelets were gated using CD41 as a platelet marker, as shown in Fig. S1 A. Platelet surface TLT-1 was significantly higher in NSCLC patients than in controls (Fig. 1 A and Fig. S1 B). The elevated TLT-1 surface levels were concurrent with an increase at the mRNA level (Fig. 1 B and Fig. S1 C) as well as at the protein level (Fig. 1 C and Fig. S1 D). While TLT-1 itself is considered a reliable and sensitive marker for platelet activation (Smith et al., 2018), we also evaluated other standard platelet surface activation markers (Kannan et al., 2019). PAC1 binding (activated α_IIb_β_3_, a platelet prothrombotic activation marker) did not change significantly in the NSCLC group (Fig. 1 D and Fig. S1 E). However, surface P selectin (granule release marker) was significantly higher in the NSCLC group (Fig. 1 E).

Given the selective increase in activation markers (P-selectin and TLT-1, but not activated α_IIb_β_3_), we proceeded to explore platelet morphology. Analysis of platelet ultrastructure further confirmed this partial activation and revealed an increased release of microvesicles from NSCLC platelets (Fig. 1 F). To further determine the functional changes in the platelets from NSCLC patients, we assessed mitochondrial depolarization and apoptosis. Unexpectedly, the NSCLC platelets demonstrated greater mitochondrial integrity and reduced apoptosis (Fig. S1, F and G). The reduced expression of activated caspase-3 and bad (pro-apoptotic) along with higher Bcl-2 (anti-apoptotic; Fig. S1, H and I) further confirmed the reduced apoptosis in NSCLC platelets.

Previously, the activation of platelets had been shown to lead to increased TLT-1 surface expression and release into circulation (Gattis et al., 2006). Consistent with the higher surface TLT-1 levels, we observed a significant elevation in the soluble form in plasma from NSCLC patients as compared to controls (Fig. 1 G). Further, surface TLT-1 was also higher in the platelet microvesicles (PMVs) in NSCLC patients (Fig. S1 J). We next investigated the relative abundance of TLT-1 in soluble form versus the microvesicle-associated form. To address this, we performed comparative analysis between circulating levels of soluble and surface-bound forms by ELISA. Interestingly, the soluble form was significantly more abundant (nearly 30 times) than the microvesicular form (Fig. S1 K). Confocal microscopy–based adhesion assays further confirmed that the activation of NSCLC platelets leads to increased expression and release of TLT-1 (Fig. 1 H). Taken together, our human platelet data reveal a unique platelet phenotype in NSCLC characterized by partial activation of platelets and increased TLT-1 expression and release.

### Platelet TLT-1 expression increases with NSCLC tumor growth in mice

**Figure 2.**
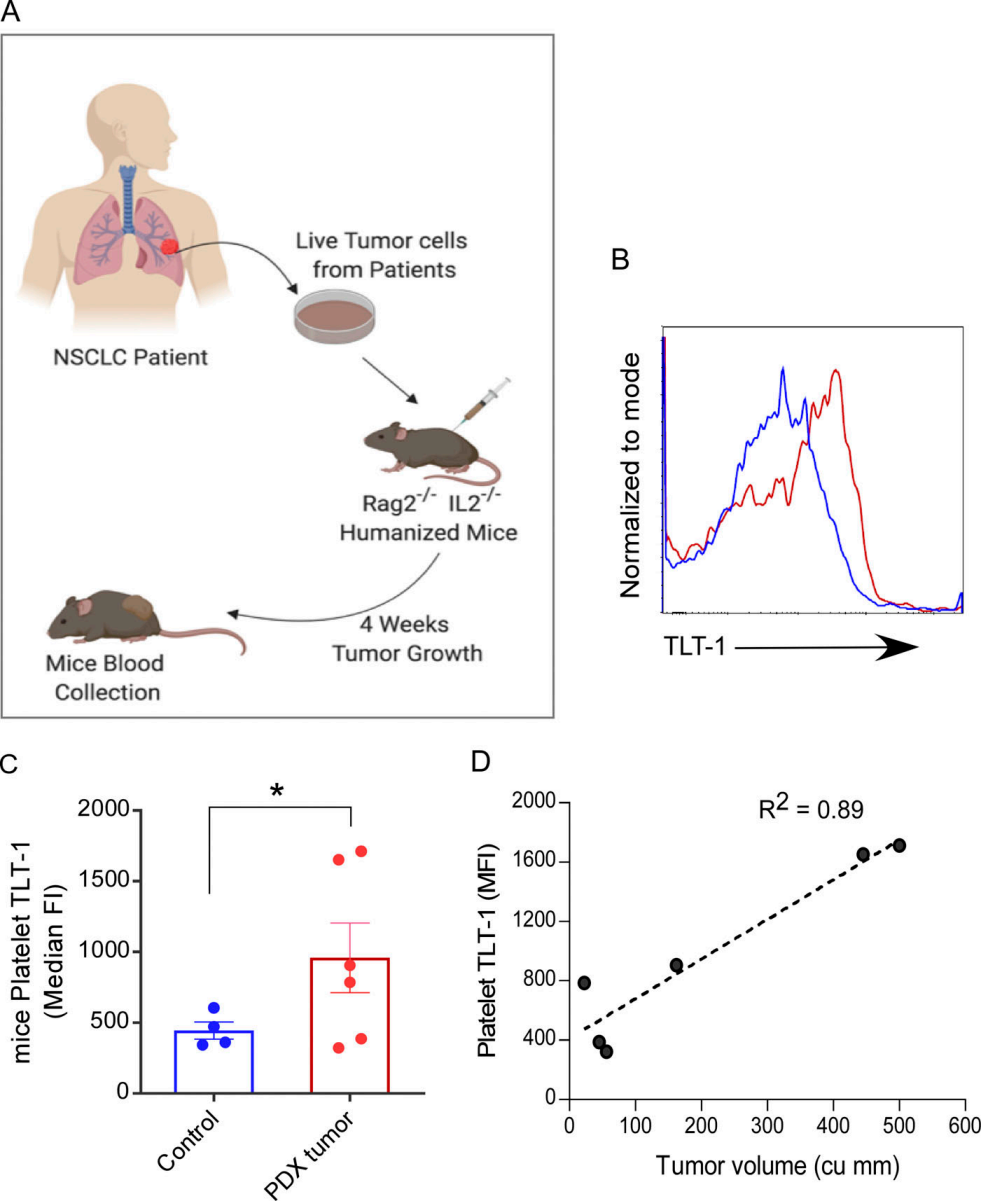
**Platelet TLT-1 expression increases with NSCLC tumor growth in mice. (A)** The immunocompromised MISTRG-6 mice (humanized) were injected subcutaneously with tumor cells derived from NSCLC patients and tumor growth was monitored for 4 wk. Platelets isolated after 4 wk from NSCLC tumor–bearing or control mice were used for FC-based assays. **(B and C)** The representative FC histogram. Blue represents control mice, red is patient-derived xenograft (PDX) tumor (B), and the quantitation (C) of surface TLT-1–staining shows increased TLT-1 staining in mice with NSCLC tumor (*n* = 4 for control, *n* = 6 for PDX tumor mice). Statistical significance tested by Mann–Whitney test. **(D)** The Pearson’s correlation curve between platelet TLT-1 level (FC-based assay shown in C) and respective tumor volume in each mouse (*n* = 6) shows significant correlation (P < 0.05). The data represent two independent experiments. The scatter plots present values with mean ± SEM. The P value shown corresponds to the applied non-parametric two tailed Mann–Whitney test. Significant differences are indicated with asterisks (*, P < 0.05).

A key question was whether the observed platelet phenotype in NSCLC patients resulted from the tumor itself or if it was secondary to comorbidities or medications/immunotherapy in these patients. To address this, tumor cells derived from NSCLC patient tumors (*n* = 6) were transplanted as xenografts into immunocompromised (*Rag2*^*−/−*^ *Il2r*^−/−^) MISTRG-6 mice (*n* = 6) in which five genes were humanized (knock-in for human *Il3*, *Thpo*, *Csf1*, *Csf2*, and *Il-6*; Rongvaux et al., 2014; Yu et al., 2017; Fig. 2 A). Platelets from mice with tumor had higher surface TLT-1 expression than those from age-matched control mice (Fig. 2, B and C). Moreover, this platelet surface expression of TLT-1 increased linearly with tumor size (R^2^ = 0.89; Fig. 2 D). These data support that the observed NSCLC-associated increase in platelet TLT-1 is independent of other factors such as comorbidities or therapies. Consistent with our human data, these results thus demonstrate that NSCLC tumor pathogenesis is associated with TLT-1 upregulation and release from platelets.

### TLT-1 injection in immunocompetent mice suppresses CD8 T cells in vivo

**Figure S2.**
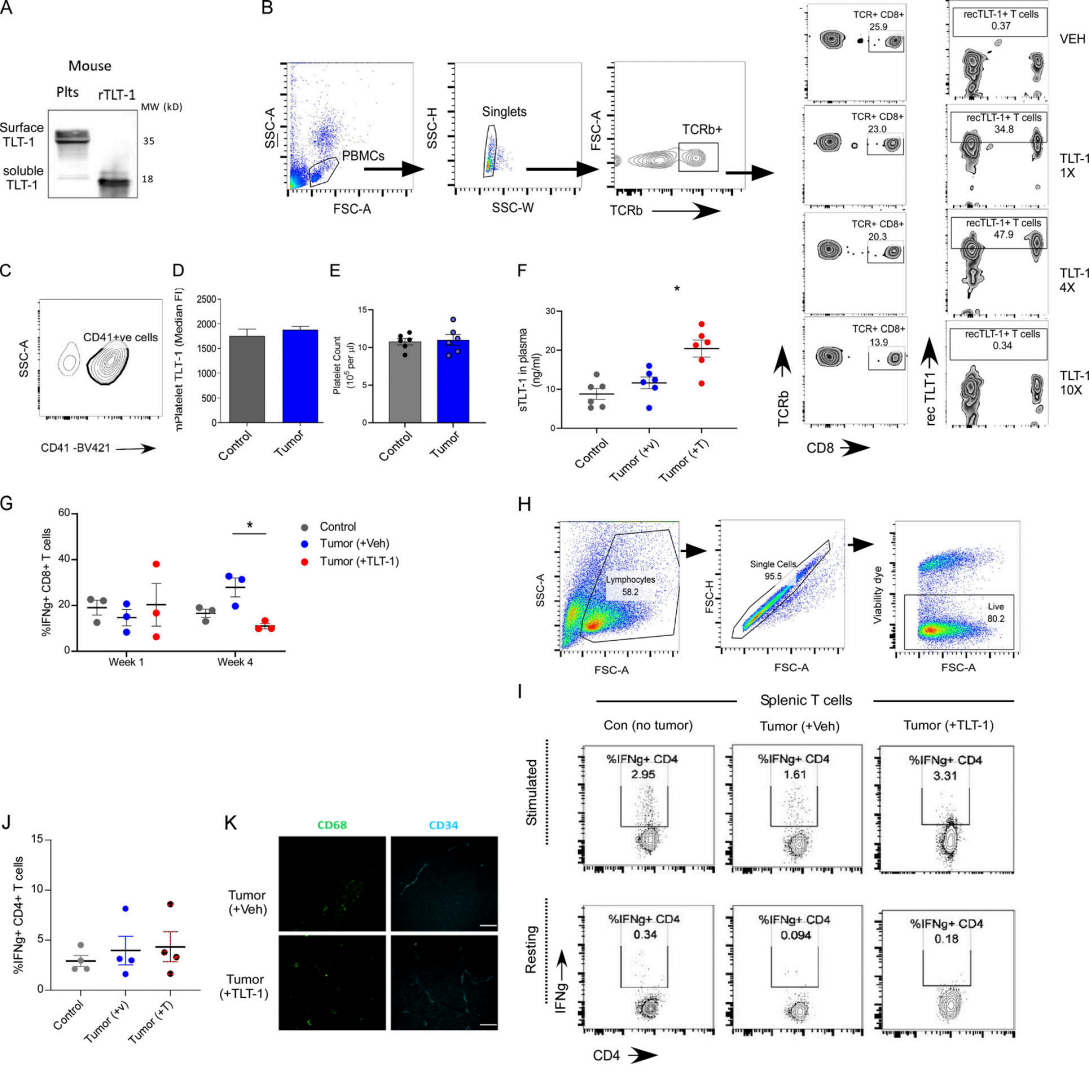
**The mouse platelet and T cell assays with recTLT-1 and B16 tumor in vivo and ex vivo. (A)** The immunoblot showing comparison of native mouse platelet TLT-1 (left lane, mouse platelets) and recTLT-1 (right lane, purified mouse TLT-1), where the sTLT-1 (left lane, soluble form, lower band) and soluble recTLT-1 give bands at the same position. This also confirms the shared antibody binding extracellular domain presence in both. **(B)** Representative FC plots for gating T cell population (left) in mouse blood and comparative FC zebra plots showing CD8^+^ T cell % of total T cells (right) and recTLT-1–bound T cell % (inside gate) in mice administered with three different amounts of recTLT-1 or Veh. The WT control mice (*n* = 4) were injected with three amounts (15, 60, or 150 µg/kg body weight: 1×, 4×, or 10× doses, respectively) and 200 μl of blood was collected after 6 h. The citrated mouse blood was processed for direct staining with conjugated antibodies for T cell (anti-mouse TCRb-FITC), CD8 (anti-mouse CD8-BV420), and recTLT-1 (anti-his tag-A-647). The recTLT-1 has a small His-tag at the C terminus, which was used for staining recTLT-1–bound cells on the surface. The cells were not lysed, and cell surface binding of recTLT-1 to T cells was analyzed. **(C)** Platelet purity and gating for platelets in freshly drawn mouse blood samples. The representative FC contour plot shows gating of CD41^+^ cells for TLT-1 expression analysis. **(D and E)** Quantitation (*n* = 6 per group) of platelet surface TLT-1 by FC assay in samples (D) or platelet counts (E; *n* = 6) from mice with (tumor) subcutaneous melanoma or without (control) after 4 wk of tumor induction. **(F)** Soluble TLT-1 quantitation as measured by ELISA after 4 wk of tumor growth. **(G)** The IFNg + CD8 T cell proportions as measured by FC before (week 1) and after (week 4) Veh or TLT-1 injections. **(H)** The representative FC gating strategy for splenic cells after isolation and culture of harvested spleens. The same strategy was followed for all the groups for spleen culture. The live cells were gated using fixable viability dye as shown. **(I)** Representative FC contour plots for resting or CD3-CD28 stimulated splenocytes gated on live CD4^+^ve cells. **(J)** The IFNg + ve cells are subgated for showing % positive CD4 T cells producing IFNg. and their mean quantitation. **(K)** The representative images (*n* = 4) show the mouse tumor cryosections immunostained by anti-CD68 and anti-CD34 (secondary Alexa-647) antibodies followed by respective secondary antibodies (Alexa-488 antibody for CD68; Alexa-647 for CD34; scale bar equals 50 μm). All the data in vertical bars shows mean ± SEM. Statistical significance tested by two-tailed unpaired Mann–Whitney, except in G where paired *t* test was applied between time points. Significant differences are indicated with asterisks (*, P < 0.05). The data are result of minimum of two independent experiments. Source data are available for this figure: SourceData FS2.

TLT-1 bears an IgV domain, which is a common feature of soluble immunocheckpoint ligands/receptors (Collins et al., 2005). With the presence of an IgV domain and extracellular release into circulation, the likelihood of TLT-1 interacting with circulating immune cells cannot be ignored. To explore this, we injected purified mouse recombinant TLT-1 (recTLT-1) protein (with intact IgV domain) into WT (immunocompetent) mice and assessed for its binding to circulating immune cells. The recTLT-1 was chosen for these mice interventional studies since it shares the complete extracellular domain sequence with the platelet-released TLT-1 form and was derived from murine cells. This was further confirmed by immunoblotting (Fig. S2 A); both the native secreted form and recTLT-1 were recognized by the same antibody and gave a distinct band at the same molecular weight, as shown in Fig. S2 A.

Based on initial optimizations which included in vitro studies and in vivo plasma TLT-1 quantitation, 100 μl of either vehicle or each of three different concentrations (15, 60, or 150 µg/kg body weight) of recTLT-1 was injected into mice via tail vein followed by flow cytometry (FC) to study peripheral blood leukocytes. Among the immune cells, we observed recTLT-1 binding to T cells in vivo (gating on recTLT-1 positive cells), thus demonstrating that extracellular TLT-1 directly interacts with T cells (Fig. S2 B). Moreover, the proportion of CD8 T cells decreased with increasing amounts of administered recTLT-1 (Fig. S2 B). These results suggest that TLT-1 is able to bind with T cells and thus can have an effect on T cell mediated antitumor immune response.

**Figure 3.**
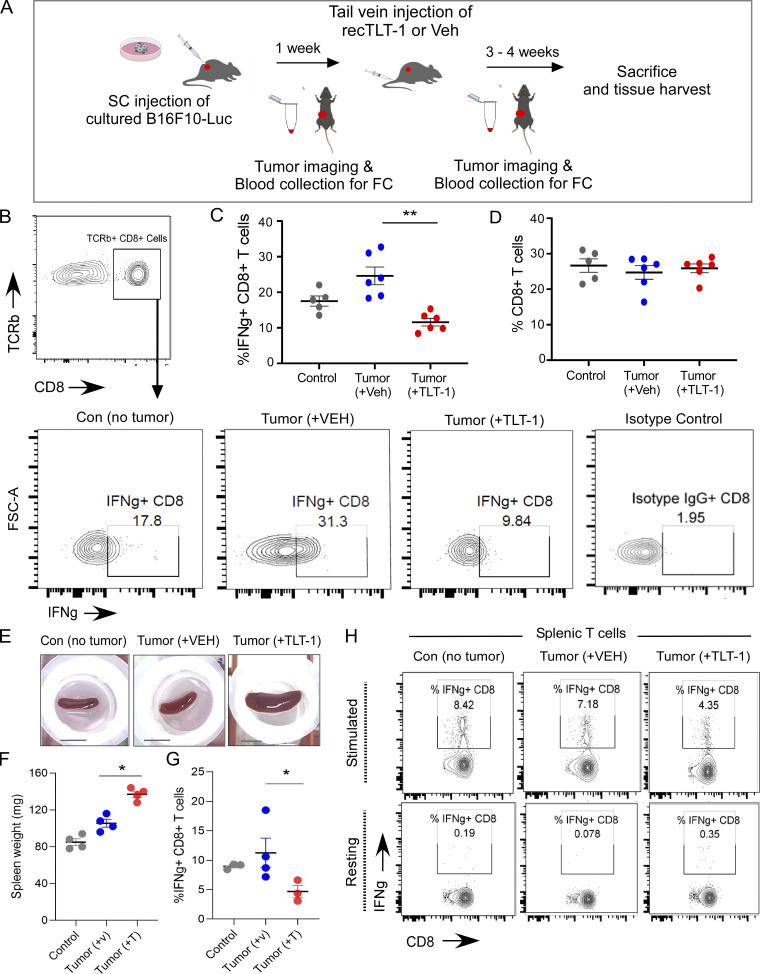
**TLT-1 injection in immunocompetent mice suppresses CD8 T cells in vivo. (A)** The subcutaneous B16F10 tumor model experiment and recTLT-1 administration strategy shown as a schematic. Age- and gender-matched control mice were injected subcutaneously with the tumor vehicle (HBSS). After 1 wk of tumor growth, one group of mice (*n* = 8) were injected with recTLT-1 once per week, while the other group (Veh, *n* = 8) received the vehicle. **(B–D)** The peripheral blood T cells (TCRb + ve) were analyzed from whole blood for circulating CD8^+^ fraction and for intracellular IFNγ levels after recTLT-1 or vehicle treatments (injections). The peripheral blood mononuclear cells from blood were stained for TCRb-FITC (T cell marker), CD8-Alexa-405, and IFNγ-PE or its isotype control-PE. The TCRb + cells were gated for CD8^+^ fractions as shown in B, and these CD8 T cells were subgated for IFNγ-positive cells in C for all three groups after treatment. The representative contour plots in B and the quantitations (C and D) are shown. Statistical significance tested by Mann–Whitney test. **(E and F)** The mice (control, tumor [VEH], or tumor [TLT-1]) were sacrificed, and harvested spleens were observed to be of different sizes as shown by whole spleen representative images (E) and weight (*n* = 4 per group; F). The scale bar equals 10 mm. Statistical significance tested by Mann–Whitney test. **(G and H)** The spleen cells were activated with anti-CD3/CD28 (H, top panel) or left unstimulated (H, bottom panel). The IFNγ-producing CD8^+^ T cells (%; G) and CD4^+^ T cells from splenic cultures were quantified, and contour plots for CD8 T cells are shown with outliers (*n* = 3–4 per group), while the CD4 T cell data are presented in Fig. S2. The FC gates show the percentage of cells, and quantitation are presented as scatter graphs with mean ± SEM and analyzed by non-parametric Mann–Whitney test or Kruskal–Wallis test as appropriate. Significant differences are indicated with asterisks (*, P < 0.05; **, P < 0.01). The data represent a minimum of three independent experiments.

Thus, we next studied the effect of the elevation of soluble TLT-1 on T cell–driven antitumor immunity in vivo using a mice tumor model with a normal functioning immune system. We injected B16F10-Fluc (firefly luciferase–expressing) tumor cells subcutaneously into WT syngeneic mice. Following tumor injection, there were no significant differences in platelet surface TLT-1 (Fig. S2, C and D) and platelet counts between control or tumor-bearing mice (Fig. S2 E). Soluble TLT-1 levels were increased in tumor-bearing mice as compared with control mice; however, unlike the NSCLC patients, this increase was not significant (Fig. S2 F). The recTLT-1 injections (via tail vein) were given after 1 wk of tumor induction (study design shown in Fig. 3 A) at a dose of 60 µg/kg body weight such that the total soluble TLT-1 levels are elevated in mice blood in the similar range as observed in NSCLC patients’ samples. As expected, the recTLT-1–injected mice had significant elevations in soluble TLT-1 (Fig. S2 F).

The circulating T cells were analyzed for the effect on numbers and activation. Previous studies have shown that IFNγ-producing T cells form the key executors of antitumor immune response in both humans and mice (Garris et al., 2018; Lin et al., 2018; Pan et al., 2018). As expected, mice with tumor (but no recTLT-1) displayed higher proportions of activated (IFNγ-producing) CD8 T cells as compared with control mice (nontumor; Fig. 3, B and C). Interestingly, the effector (IFNγ-producing) CD8 circulatory T cells were significantly lower in the tumor-bearing recTLT-1 mice than in the tumor-bearing vehicle group (Fig. 3, B and C). This approach provides direct evidence for T cell targeted immunomodulatory effects of extracellular TLT-1 elevation in blood.

The proportions of circulating CD8 T cells were not significantly different between the groups (Fig. 3 D), which could be due to the recovery of T cells in blood, days after injection. This was different from what we observed in nontumor mice after 6 h of recTLT-1 injection (Fig. S2 B), likely due to the difference in the time point of measurement after injection. The analysis was further extended to compare T cells at week 1 and 4 of tumor growth i.e., before and after TLT-1/vehicle injections in mice, which further confirmed the decrease in effector (IFNγ-producing) CD8 T cells in TLT-1–injected mice (Fig. S2 G). Further, we observed splenomegaly (without splenic tumor invasion) in all the tumor-bearing mice (Fig. 3, E and F). This is consistent with previous reports demonstrating enlarged spleens in the later stages of tumor growth (Miluzio et al., 2011). Interestingly, the mice that received recTLT-1 showed splenomegaly with significantly greater spleen weights than the other two groups (Fig. 3, E and F). To further analyze the effect of higher TLT-1 on T cell activation, we performed splenic T cell culture from these mice. We tested T cell activation of splenic cells to understand the effect of injected TLT-1 (in vivo) on T cells upon in vitro stimulation. The IFNγ-producing CD8^+^ fractions were significantly reduced in recTLT-1–injected group as compared with the vehicle group of mice upon in vitro stimulation by stimulatory (anti-CD3/CD28) antibodies (Fig. 3, G and H; and Fig. S2 H). However, the CD4^+^ splenic T cells did not show a reduction in IFNγ+ve fractions (Fig. S2, H–J), suggesting a suppressive effect of TLT-1 elevation selectively on effector CD8 T cells but not on CD4 T cells. This also suggests that the T cell suppressive action of TLT-1 on CD8 T cells is not limited to the continued exposure to TLT-1 in circulation. Altogether, our data provide evidence for interaction between TLT-1 and T cells and a strong suppressive effect on effector CD8 T cells in response to elevated extracellular TLT-1 in vivo.

### Elevated TLT-1 in circulation promotes tumor progression in mice

**Figure 4.**
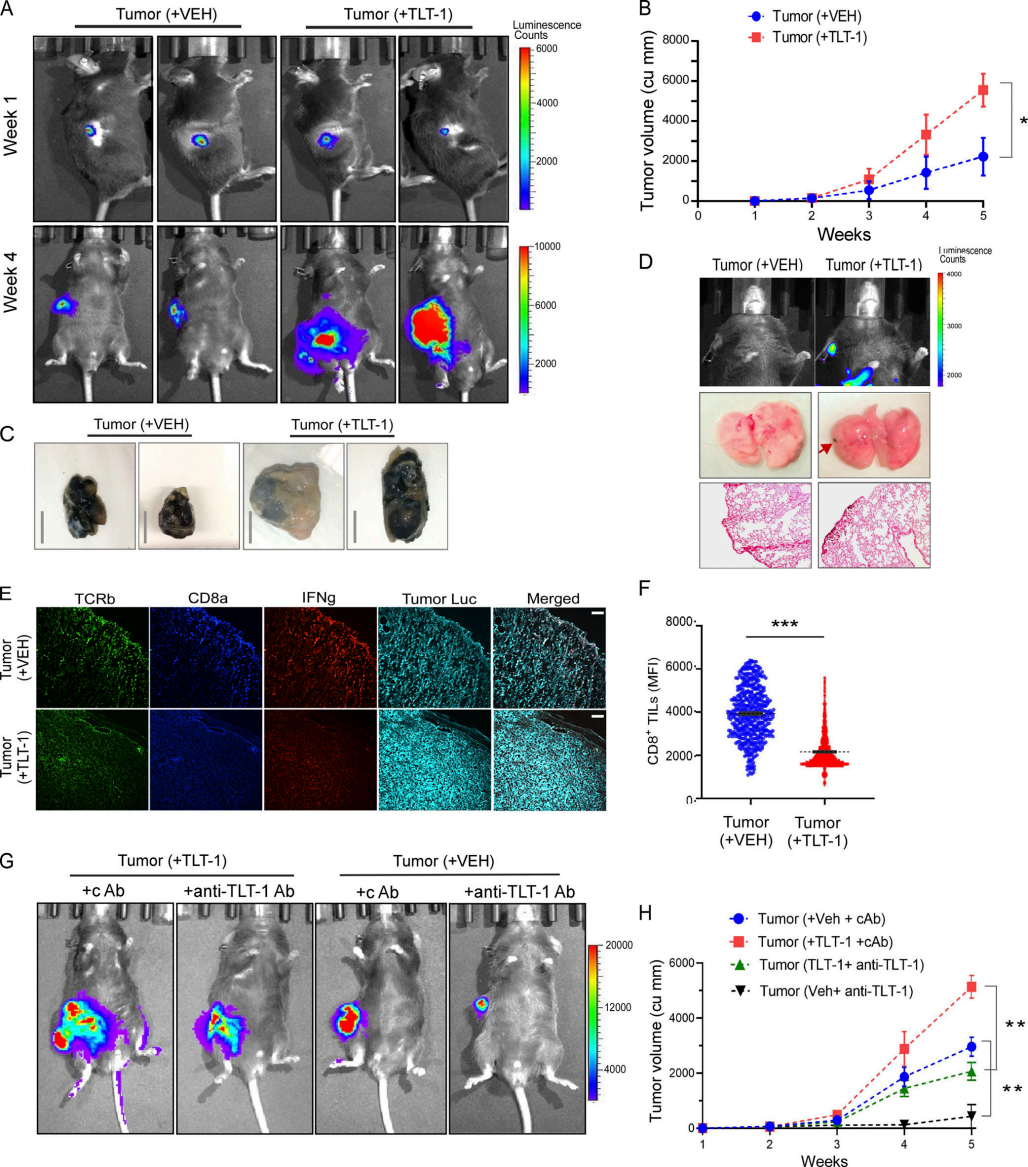
**Elevated TLT-1 in circulation promotes tumor progression in mice. (A)** Representative images of in vivo imaging of luciferase-labeled tumor in mice before (upper panel) and after (lower panel) recTLT-1 or vehicle administration. **(B)** Quantified weekly measured tumor sizes (volume) are shown for mice induced with tumor and compared between Veh and TLT-1 administered mice (*n* = 8 per group). The data from both groups at multiple time points were analyzed by repeated two-way ANOVA. **(C)** Representative images of primary tumors excised from mice for both treatment groups (scale bar = 10 mm). **(D)** Representative bioluminescence imaging of thoracic region of mice bearing primary subcutaneous tumor after 5 wk of tumor growth (+VEH or +TLT-1) showing metastatic spread of tumor in recTLT-1–injected mice (top panel). The images were captured using PerkinElmer’s in vivo imaging system (IVIS) immediately after luciferin injection (i.p.). The images of excised lungs (upper panel) from mice with or without tumors showing metastasis in recTLT-1–administered group (middle panel). The respective Fontana-Masson–stained sections (original magnification, 100×) are shown to confirm the tumor presence as the black region on the peripheral side of lungs in the recTLT-1 group (bottom panel). **(E and F)** Representative tumor tissue micrographs (immunofluorescence) compare the CD8^+^ TILs and IFNγ expression, towards the tumor periphery, showing reduced CD8^+^ TILs and IFNγ in recTLT-1–treated mice, and CD8^+^ TILs quantitation (F) is shown. Statistical significance tested by Mann–Whitney test. The anti-luciferase antibody was stained to identify tumor cells (cyan). **(G)** Representative images (*n* = 5) of in vivo imaging of luciferase-labeled tumor in mice after 4 wk of indicated administration, which started after 7 d of subcutaneous B16F10 tumor injection. **(H)** Quantified weekly measured tumor sizes (volume) are shown for mice induced with tumor and the indicated treatment (Veh or recTLT-1 with cAb or anti–TLT-1 antibody) and compared between groups. The data from all four groups (*n* = 5 per group) at multiple time points were analyzed by repeated two-way ANOVA. The quantitation is presented as mean ± SEM, and the P values correspond to Mann–Whitney test or repeated two-way ANOVA as appropriate. Significant differences are indicated with asterisks (*, P < 0.05; **, P < 0.01; ***, P < 0.001). All the data represents three independent experiments.

**Figure S3.**
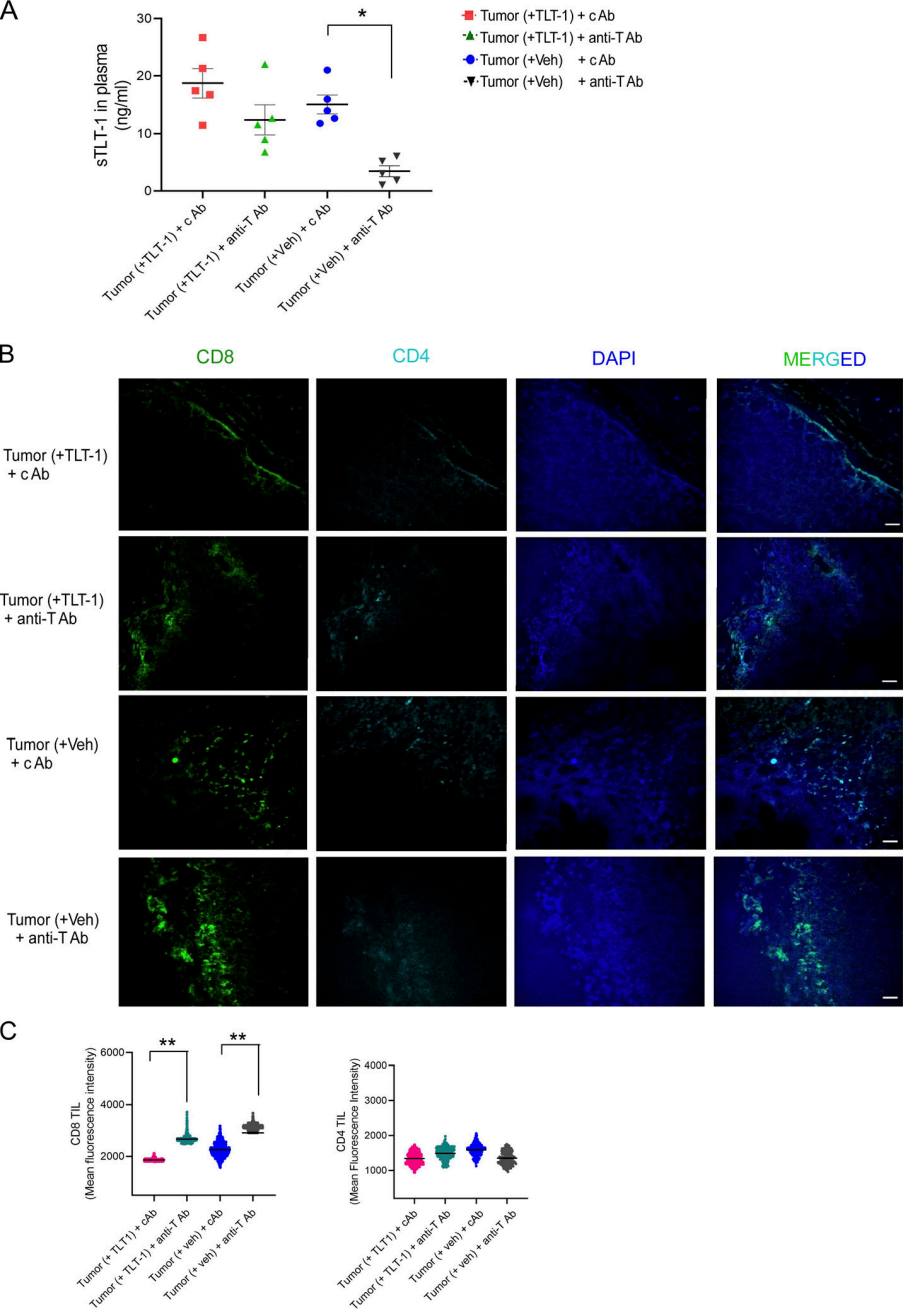
**Administration of anti–TLT-1 antibody substantially reduces tumor growth in mice. (A)** Quantitation of soluble TLT-1 in mouse plasma as measured by ELISA in all four study groups. **(B and C)** Representative mouse tumor immunofluorescence confocal images (*n* = 5 biological samples with at least 10 micrographs captured per section). The scale bar equals 50 µm. The mouse tumor tissue invasive margins showing CD8 (column first from left) and CD4 (second from left) in four study groups with significant infiltration of CD8 TILs in tumor (Veh) + anti–TLT-1 antibody group, and the quantitation (C) of mean intensity from samples of each group quantified together for intergroup comparisons. The scatter plots show mean ± SEM analysed by Kruskal–Wallis test with Dunn’s multiple comparisons. Significant differences are indicated with asterisks (*, P < 0.05; **, P < 0.01). The data represent the results of two independent experiments.

Next, we examined whether the immunosuppressive effect of TLT-1 elevation in mice is translated into tumor progression in B16F10-challenged mice (as depicted in Fig. 3 A). The in vivo imaging of luciferase-expressing tumors allowed the visualization of tumor progression before and after the recTLT-1 or vehicle administration (Fig. 4 A). The measurement of tumor size revealed that the growth of the primary tumor occurred at a faster rate in the recTLT-1–injected mice (*n* = 8) than in the vehicle group (*n* = 8; Fig. 4, B and C). Though lung metastasis rates are recognized to be poor in subcutaneous tumor models, in two of the recTLT-1–injected mice, lung metastasis was observed (Fig. 4 D, upper panel). This was also confirmed by melanin staining (Fig. 4 D, lower panel), which was negative in the vehicle group. Next, we analyzed the effect on tumor-infiltrating T cells (CD8 and CD4 TILs) by immunofluorescence microscopy. The tumors showed reduced CD8 T cell infiltration (CD8 TILs) and lower IFNγ in the recTLT-1–injected mice (Fig. 4, E and F), supporting increased tumor growth and compromised antitumor response by CD8 T cells in these mice. The CD4 T cell infiltration did not differ in recTLT-1–injected mice as shown (Fig. S3, B and C). This selective effect of elevated soluble TLT-1 on CD8 T cells but not on CD4 T cells is consistent with our splenic T cell stimulation assays (Fig. 3, G and H; and Fig. S2, H–J). We also assessed other parameters which could affect tumor growth, such as tumor macrophages and vascularity. The analysis of macrophage (CD68 marker) and vascular density (CD34 marker) in tumors revealed no apparent differences between Veh- and TLT-1–injected mice (Fig. S2 K). All these findings further highlight the importance of soluble TLT-1 for antitumor response of CD8 T cells. Taken together, these results demonstrate that elevated TLT-1 in vivo accelerates tumor progression and leads to the suppression of activated CD8 T cells in tumor.

### Administration of anti–TLT-1 antibody significantly reduces tumor growth in mice

To test the therapeutic potential of TLT-1 inhibition in vivo and to better understand the effect of inhibiting host platelet-derived TLT-1, we administered anti–TLT-1 antibody (binding to IgV domain region of TLT-1) in mice with syngeneic B16 tumors. After the initial growth of tumors, from day 7 onwards, mice with similar tumor growths were randomly divided into four subgroups. Two subgroups were administered recTLT-1 and the other two were given vehicle (Veh) as described in Fig. 3 A. Both recTLT-1 and Veh groups were administered with either anti–TLT-1 antibody or IgG isotype (control antibody [cAb]) at a fixed dose of 0.45 mg/kg body weight per mouse once every 5 d, starting from day 7 after tumor injection. The anti–TLT-1 antibody-injected mice group showed a substantial reduction of tumor growth (Fig. 4, G and H). This was accompanied by a significant reduction also in soluble TLT-1 levels in these mice (Fig. S3 A), thus providing validation for a key role of TLT-1 in promoting tumor progression.

The role of T cells in mediating this effect was assessed by analyzing tumor infiltration of T cells. CD8 TILs were significantly increased in the anti–TLT-1 antibody groups (P = 0.005 and P = 0.01) as compared with cAb groups (Fig. S3, B and C), while CD4 TILs were marginally reduced. Taken together, these experiments also suggest the importance of soluble TLT-1 for tumor progression. Moreover, the inhibition of TLT-1, whether it is injected recTLT-1 or platelet-derived soluble TLT-1, leads to a strong antitumor response. As, the tumor-bearing mice administered with TLT-1 blocking antibody (without recTLT-1 presence), which thus targets native mouse TLT-1 in vivo, showed a significant reduction in tumor size, this further emphasizes the importance of TLT-1 in tumor progression. All these data not only further support our other in vivo results but also support targeting TLT-1 as a potential antitumor therapeutic strategy, which, as we demonstrated, can be achieved by using TLT-1–specific antibody.

### Higher TLT-1 expression is associated with reduced survival of NSCLC patients and CD8 T cell exhaustion in tumors

**Figure 5.**
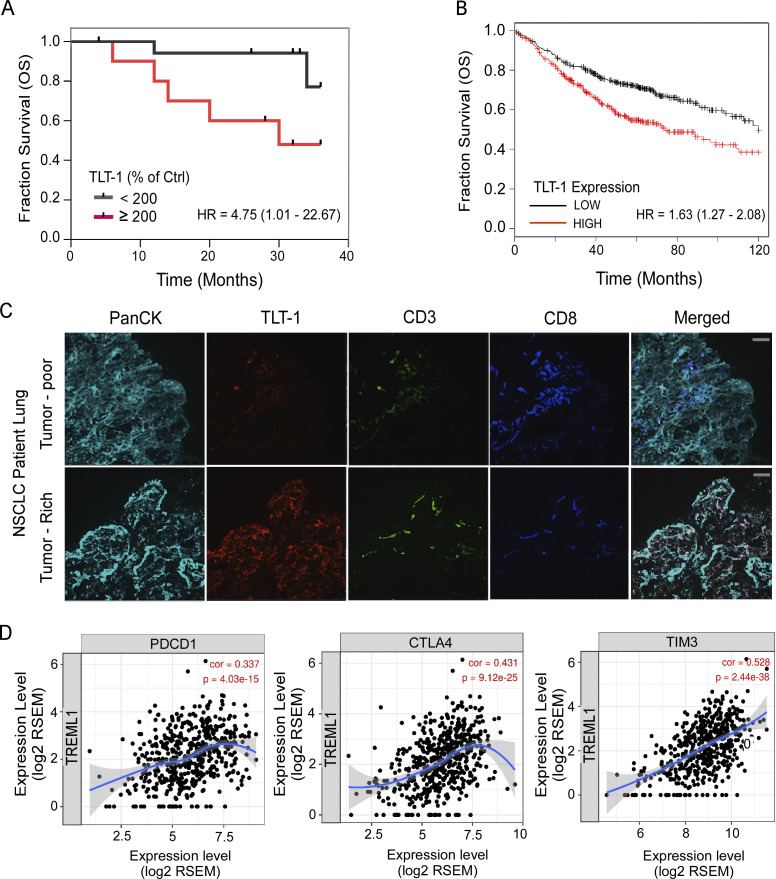
**Higher TLT-1 expression is associated with reduced survival of NSCLC patients and tumor CD8 T cell exhaustion. (A)** The survival curves (KM-plot) for the present study cohort showing significant difference (logrank Mantel–Cox P value = 0.036) between OS of patients with very high (≥200) platelet surface TLT-1 expression as measured by FC (normalized as % change over control) as compared to those with relatively lower levels (<200). The 200 cut-off was chosen because the median percent increase for these patients was very close (190) to this value. The plot shows follow up of a maximum of 3 yr for this cohort (logrank Mantel–Cox test). **(B)** The KM-plots for median 10-yr OS survival of NSCLC patients plotted with TLT-1 expression are shown as extracted from the KM plotter database, showing significantly (P < 0.0001) reduced OS. The details of patient cohorts used for generating the presented survival curve (B) are given in Fig. S5 B. The logrank test hazard ratio (HR) values along with 95% confidence interval range shown for each plot. **(C)** The NSCLC patient lung sections (representative for *n* = 6, with 6–10 frames per section) with positive TLT-1 staining (red) showing increased TLT-1 and reduced CD8 T cells (violet) in tumor-rich areas as compared to the tumor-poor areas from lung biopsy samples of same patient. The PanCK marker (cyan) was used for identifying tumor cells; CD3 (green) was used as a T cell marker. The original magnification was 200×, and the scale bar equals 100 µm. **(D)** TIMER, a webtool, was used to assess the relationship between TLT-1 and CD8 T cell infiltration in lung adenocarcinoma tumors using previous high-throughput gene datasets. The TLT-1 expression shows significant correlations with T cell exhaustion markers—CTLA-4, PDCD-1 (PD-1), and TIM-3 in tumor microenvironment. The correlation coefficient and P values are shown. The experimental data represent a minimum of two independent experiments.

**Figure S4.**
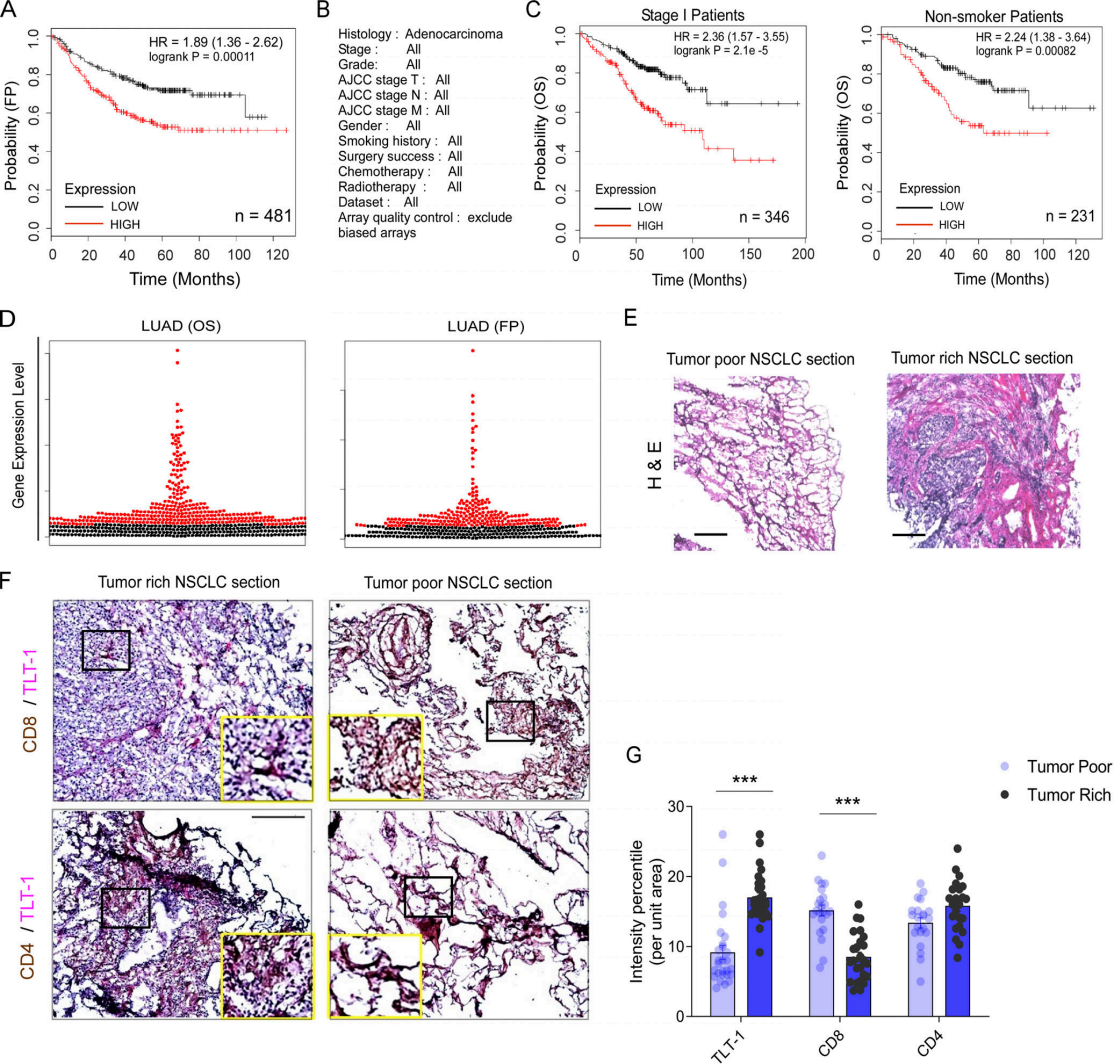
**The KM-plots for survival and lung histology of NSCLC patients. (A and B)** The association of TLT-1 expression with reduced survival in NSCLC and patient subgroups. The FP survival curves for TLT-1 expression extracted from various previous microarray studies for all stage NSCLC patients (LUAD), with hazard ratios and P values as specified. **(C)** The OS KM-plot for early stage (TNM stage 1) on the left and for non-smokers on the right. **(D)** The beeswarm plots for all NSCLC patients whose survival curves are shown. The red dots are the samples categorized as “high expression,” while the black dots are the “low expression” category for TLT-1, as per the analysis from the used database. **(E)** H&E-stained cryosections of human NSCLC tumors. Scale bar represents 100 µm. **(F and G)** The representative images (F) from dual IHC assay on NSCLC cryosections of tumor-rich or tumor-poor samples showing CD8/TLT-1 staining (top panels) or CD4/TLT-1 staining (bottom panels). The CD4 and CD8 stainings are colored brown, while TLT-1 is represented by pink stain. The slides were counterstained by Gill’s hematoxylin (blue). Zoom in on the square insets shown at either corner in yellow boxes (scale bar = 200 μm) for enhanced clarity. The respective stained areas were quantified (G) in tumor areas (excluding stromal regions; four to eight frames per section) by an observer blinded to experimental groups and are presented as mean ± SEM. Significant differences are indicated with asterisks (*, P < 0.05; **, P < 0.01; ***, P < 0.001). The data represent the results of two independent experiments.

We next evaluated the clinical potential of TLT-1 in NSCLC progression and prognosis. In our patient cohort, we recorded the survival time span using a Kaplan–Meier plot (KM-plot) based on platelet TLT-1 levels as measured by us (Fig. 5 A). High-platelet TLT-1 was associated with reduced overall patient survival, as measured in our cohort (Fig. 5 A). To further confirm and validate the association of TLT-1 expression with NSCLC patient survival in larger cohorts, we used an open cancer patient webtool that utilizes previously submitted gene expression datasets from cohorts and provides data with appropriate quality control (Lanczky et al., 2016). The KM-plots from NSCLC cohorts (adenocarcinoma, all stages, *n* = 720) showed significantly decreased overall survival (OS) and free of progression (FP) survival with higher expression of TLT-1 (Fig. 5 B and Fig. S4, A and B). The median survival (OS) with higher TLT-1 was 74 mo as compared with 119 mo for patients with lower TLT-1 (Fig. 5 B). Early-stage NSCLC patients (*n* = 346) as well as non-smokers (*n* = 231) also showed a negative association with survival with higher TLT-1 expression (Fig. S4 C). The beeswarm curve for TLT-1 expression data for the KM-plots from databases are also provided (Fig. S4 D).

To add further insights into the effects of TLT-1 presence in NSCLC patient lungs, we stained cryosections from human NSCLC biopsy samples. Positive TLT-1 staining was observed in four of the six sections with increased TLT-1 in tumor-rich sections as compared to tumor-poor regions of the same patient (Fig. 5 C). There was a reduced presence of CD8 T cells in the tumor-rich areas as compared with tumor-poor sections. The corresponding H&E-stained sections are also shown to confirm tumor histology (Fig. S4 E). To further confirm CD8 as well as CD4 T cell infiltration levels in human tumors, dual IHC staining was performed. The tumor-rich sections showed increased TLT-1 and reduced CD8 staining as compared to sections with poor tumor density; however, CD4 T cells appeared to have the opposite pattern (Fig. S4, F and G). This inverse correlation of TLT-1 and CD8 T cell traces in the tumor microenvironment further supports our mice data which demonstrated the tumor-promoting and CD8 T cells suppressive effects of TLT-1.

Next, we studied the correlations between expression of TLT-1 and T cell exhaustion markers such as CTLA-4, PD-1, and TIM-3 in lung tumors using a data mining approach (Li et al., 2017; Fig. 5 D). There were significant positive correlations between TLT-1 and CTLA-4, PD1 (PDCD1), or TIM-3 in the tumor microenvironment, emphasizing the association of TLT-1 with CD8 T cell exhaustion in tumors. Taken together, the data from our patient cohort and the computational analyses of datasets from previous cohorts support that the higher platelet TLT-1 is associated with poor clinical outcome in NSCLC patients; moreover, TLT-1 likely plays an important tumor-promoting role in NSCLC tumor microenvironments.

### TLT-1 binds to CD3ε on T cells and suppresses CD8 T cells but not CD4 T cells

**Figure 6.**
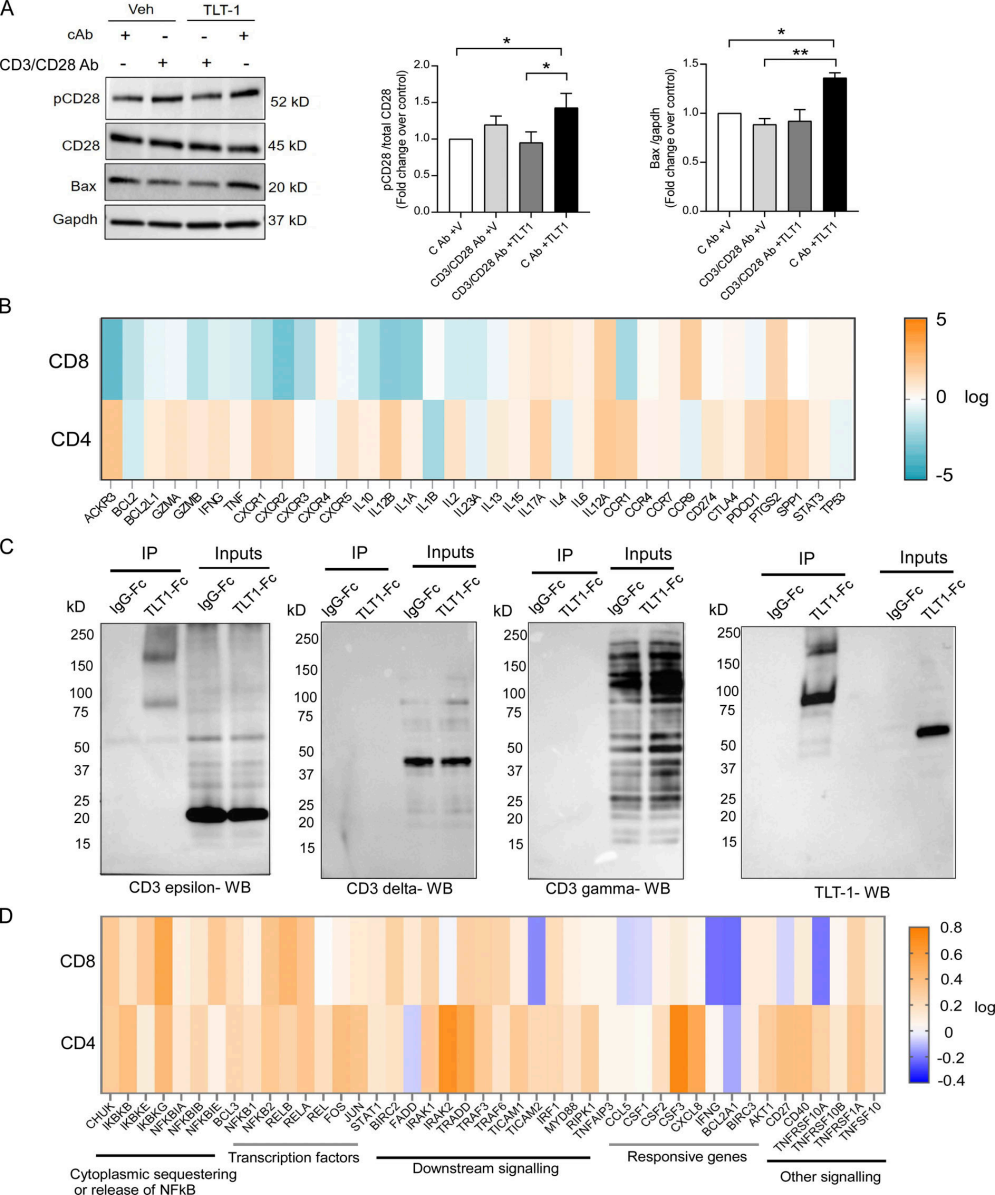
**TLT-1 binds to CD3ε on T cells and suppresses CD8 T cells but not CD4 T cells. (A)** The initial functional changes in primary human T cells incubated with recTLT-1 was studied by evaluating the expression of p-CD28 and Bax, as shown by representative Western blots (left) and their quantitation (right). **(B)** The heatmap obtained by qPCR array shows recTLT-1–induced gene regulation (arithmetic mean) in CD4 and CD8 T cells isolated from healthy subjects (*n* = 4). The fold regulation (scale on the right) shows fold change upon recTLT-1 exposure for 24 h in culture with respect to vehicle control group. The qPCR dCt values were using a mean dCt of five constitutive genes as per recommendation of the array manufacturer. **(C)** Co-IP experiment using Jurkat cells incubated with Fc-tagged recTLT-1 or recIgG followed by Protein A/G bead pull down and probed with CD3ε, TLT-1, CD3γ, or CD3δ antibodies separately as shown by representative Western blots (*n* = 4 for each Co-IP). The band for CD3ε is observed at the position of TLT-1 in IP samples. The representative blots showed the higher (than in non-IP lysate) molecular weight band at ∼80–85 and ∼160–180 kD in immunoblotting of CD3ε (predicted ∼23 kD), and also when probed for TLT-1 on separate blot (predicted ∼55 kD) only in the co-IP sample. **(D)** The gene expression heat map for NF-κB pathway showing the fold change upon recTLT-1 exposure for 24 h in culture with respect to vehicle control group. The qPCR dCt values were using a mean dCt of five constitutive genes as per recommendation of the array manufacturer. We combined array data from independent experiments to select a consensus set of regulated genes. Significant differences are indicated with asterisks (*, P < 0.05; **, P < 0.01). All experimental data represent a minimum of three independent experiments. Source data are available for this figure: SourceData F6.

The next critical question was identifying the target and mechanism of action of TLT-1 on T cells. We began by assessing whether TLT-1 affects CD3/CD28-mediated activation of T cells. We stimulated freshly isolated human T cells with CD3/CD28 stimulatory antibodies, with or without recTLT-1, and evaluated CD28 phosphorylation as it initiates intracellular T cell activation events (Pages et al., 1994). In the presence of TLT-1, phosphorylated CD28 (p-CD28) did not change significantly in stimulated T cells (Fig. 6 A). However, treatment with TLT-1 in unstimulated cells (cAb) led to increased p-CD28 expression (Fig. 6 A), suggesting T cell hyperactivation by TLT-1. T cell hyperactivation can also promote apoptotic cell death, with Bax expression (in T cells) being one of the critical mediators (Bouillet and Strasser, 2002). Bax expression (pro-apoptotic marker) was significantly elevated in T cells treated with TLT-1 (Fig. 6 A).

To better understand how platelet-derived TLT-1 affects the activation-associated molecular profile of healthy human T cells, we cultured CD4 and CD8 T cells from healthy subjects in the presence of either purified active human recTLT-1 or the respective vehicle and assessed the expression profiles of major cytokines and other functionally relevant genes using a targeted gene array (Fig. 6 B). TLT-1 treatment downregulated (>1.5 fold) genes that promote survival (*BCL2*, *BCL2L1*, and *ACKR3*) in CD8 but not in CD4 T cells. Moreover, the activation-promoting genes (*GZMB*, *IL2*, *IL12B*, *IFNG*, and *TNF*) involved in antitumor responses and the CXCR family genes (*CXCR1*, *CXCR2*, and *CXCR3*) associated with improving antitumor responses by effector T cells (Kurachi et al., 2011; Peng et al., 2010; Takata et al., 2004) were also downregulated in treated CD8 T cells. These results demonstrate that the suppressive effect of TLT-1 on CD8 T cells is reflected in their transcriptome. This dataset also highlights the differential effect of TLT-1 on CD4 and CD8 T cells.

**Figure S5.**
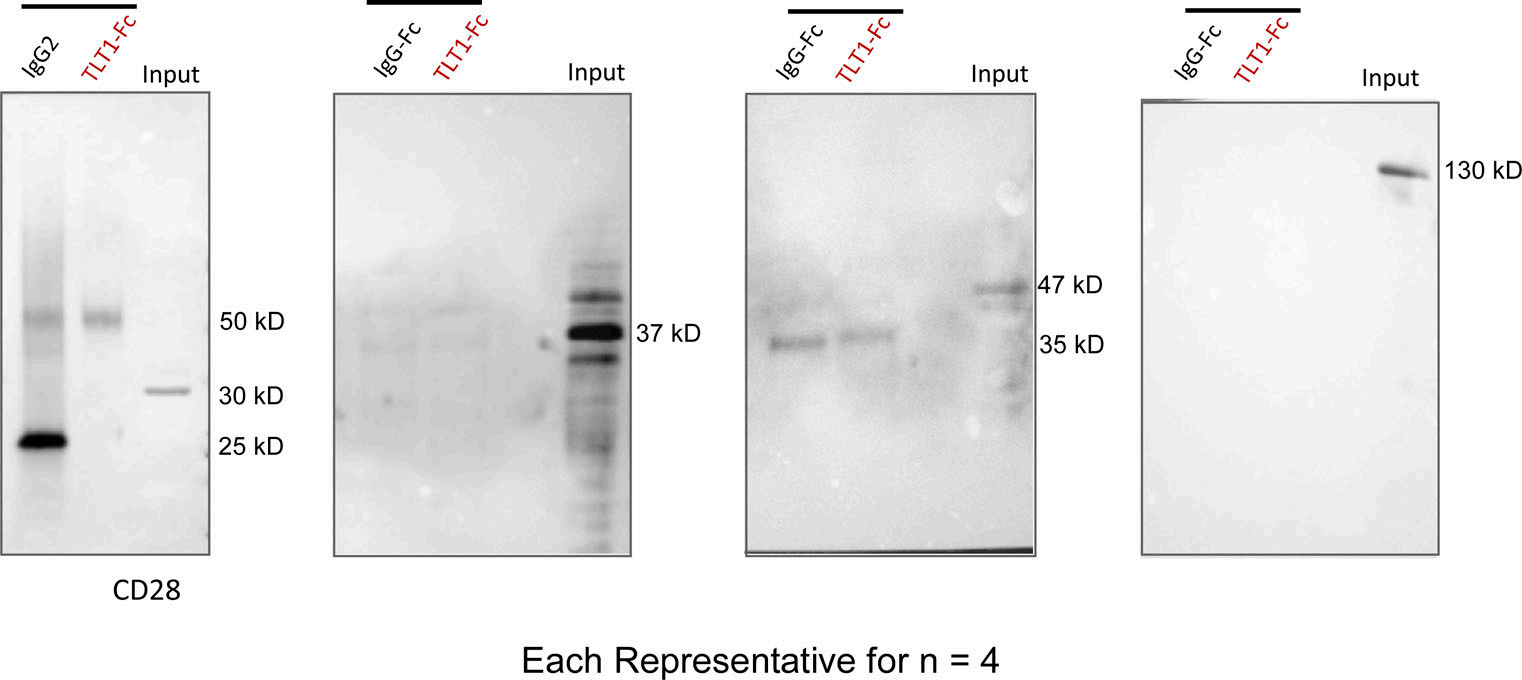
**TLT-1 Co-IP assay for undetected receptors.** The representative Co-IP pull down Western blots showing negative signal for CD28, TNFR, TRAILR3, and TRAILR2 (three independent experiments, *n* = 4 per group). The band size indicates approximate molecular weights estimated by standard molecular weight markers. Source data are available for this figure: SourceData FS5.

To identify the receptor for TLT-1 on T cells, we performed coimmunoprecipitation (Co-IP) studies. Jurkat cells (human-derived T-lymphocyte cell line) were incubated with human recTLT-1, followed by recTLT-1 pulldown, and then probed for the candidate receptors. As our previous data suggested an activation-induced cell death (AICD)–like response, we first probed for T cell surface receptors such as TNFR, TRAILR, and CD3 subunits, which are known to induce the AICD (Fig. S5). Among all these receptors, CD3 was strongly detected in the Co-IP pull-down assay. However, only the ε subunit of the CD3 receptor (CD3ε) gave a reproducible strong band/signal, while other subunits CD3γ or CD3δ present on the T cell surface were not detectable after Co-IP (Fig. 6 C). The Co-IP of TLT-1 was negative for CD28 (Fig. S5). The interaction between CD3ε and TLT-1 was confirmed by using antibodies from multiple sources. This suggested that TLT-1 specifically binds CD3ε on T cells.

CD3 is also known to trigger AICD upon reactivation of CD8 T cells which involves the NF-κB pathway (Huang et al., 2018; Shi et al., 1990). Therefore, we next carried out NF-κB–targeted gene array analysis of primary human T cells cultured with or without recTLT-1. We found remarkable changes in expression levels of NF-κB pathway genes including genes regulating the sequestering of NF-κB proteins (*IKBKE*, *IKBKG*, and *NFKBIE*), transcription factors (*NFKB2*, *REL*, *FOS*, and *JUN*), downstream signaling (*IRAK2*, *TRADD*, *TRAF2*, *TBK1*, etc.), and responsive genes (*IFNγ*, *CSF3*, and *BCL2A1*; Fig. 6 D). While *FOS* (*c-Fos*) and *JUN* (*c-Jun*) are known to induce T cell activation (Ullman et al., 1990), *FOS* previously has been shown to suppress T cells through PDCD1 and promote tumor progression (Xiao et al., 2012). More in-depth studies are needed to elucidate the precise underlying signaling mechanisms.

Thus, we here identify CD3ε on T cells as a receptor for TLT-1 and found the suppression of CD8 T cells to involve the NF-κB pathway. Taken together, these results suggest an activation-associated apoptosis process or an AICD-like phenotype in CD8 T cells in response to TLT-1.

### Anti–TLT-1 antibody prevents the platelet-induced suppression of patient CD8 T cells

**Figure 7.**
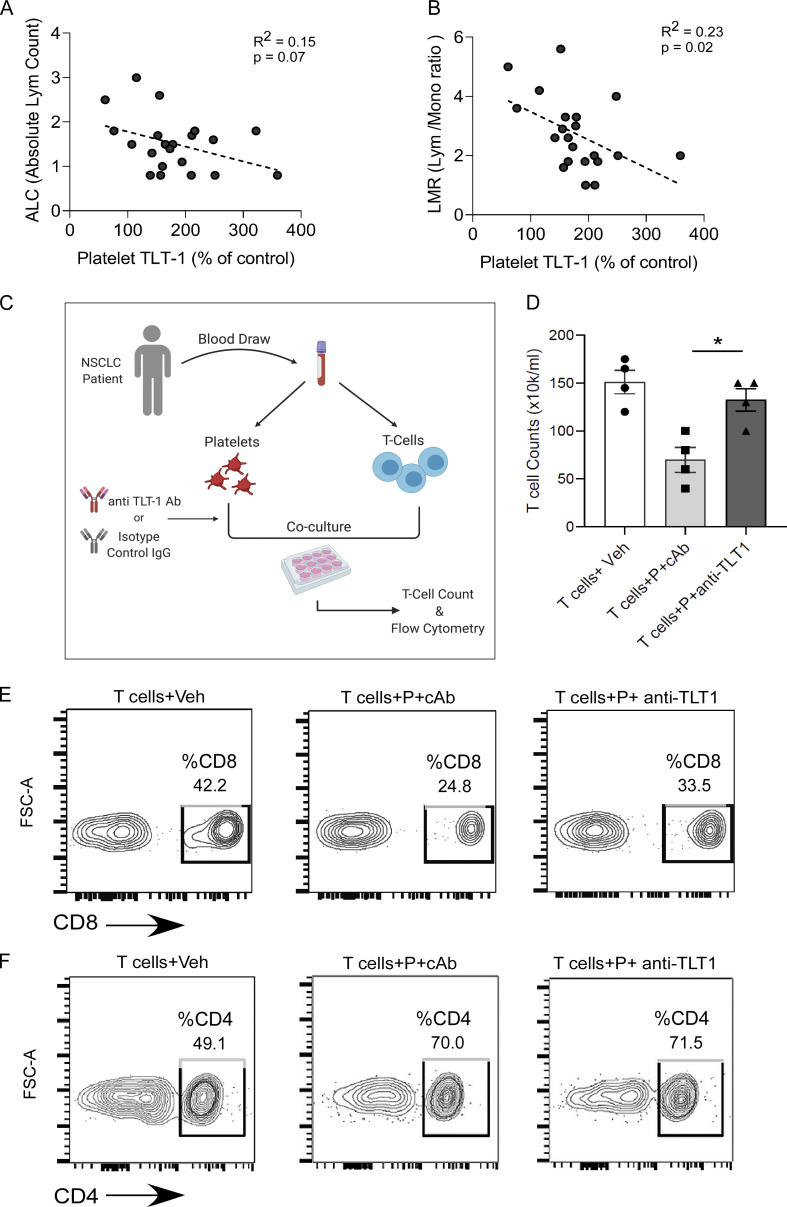
**Anti–TLT-1 antibody prevents the platelet-induced suppression of patient CD8 T cells. (A and B)** The clinical profiles of studied NSCLC patients were screened for hematology reports and absolute lymphocyte count (A) as well as LMR (B) were recorded for individual patients at the closest date to recruitment and sampling. The obtained values were plotted as x*–*y correlation plots (A and B) with surface TLT-1 levels measured in those patients. An inverse relationship of surface TLT-1 was found with both these lymphocyte-based parameters. The Pearson’s coefficient is shown. **(C)** The schematic shows the experimental strategy followed for studying the role of platelet-released TLT-1 in T cell exhaustion by coculture of NSCLC patient platelets with T cells. The magnetic bead–based negative selection kit was used to isolate T cells from patient blood. **(D)** Live T cell numbers after coculture of platelets with T cells from NSCLC patients in presence of anti–TLT-1 antibody or isotype control (cAb) antibody. A control group with NSCLC T cells only and no platelets is also shown. Quantitations are presented as scatter graphs with mean ± SEM, and the significant differences indicated by asterisk correspond to Kruskal–Wallis test followed by Dunn’s multiple comparisons. **(E and F)** The FC-based analysis of T cells (*n* = 4) from coculture experiment shows the fraction of CD8 cells (layout gates), or CD4 T cells (F) in the three groups. The representative contour plots (*n* = 4) were the result of gating on lymphocyte proportion. The FC gates show the % cells. Significant differences are indicated with asterisks (*, P < 0.05). All experimental data represent minimum of three independent experiments.

Based on our findings, we next sought to validate the CD8 T cell suppressive activity of elevated platelet TLT-1 in NSCLC patients. We initially assessed whether the TLT-1 increase on circulating NSCLC platelets was associated with immune hematological parameters, such as lymphocyte counts and the lymphocyte-to-monocyte ratio (LMR). LMR was chosen as a lymphocyte-based biomarker that is also a recognized independent prognostic marker for NSCLC from multiple clinical studies, and its decrease is associated with poor prognosis (Lin et al., 2014; Teng et al., 2016). We observed a significant negative correlation between platelet surface TLT-1 and LMR, and a similar trend was observed with absolute lymphocyte counts (Fig. 7, A and B). These data suggested that increased platelet TLT-1 is associated with overall negative prognostic lymphocyte markers in NSCLC patients.

To test the effect of TLT-1 inhibition on T cells in patient samples, we designed an ex vivo platelet–T cell interaction study in which we simultaneously isolated platelets and T cells from the same patient (autologous). The T cells were cocultured for 48 h with autologous platelets and pretreated either with anti–TLT-1 antibody (T cells + P + anti–TLT-1) or an isotype cAb (T cells + P + cAb; as described in schematic Fig. 7 C). A coculture control group (T cells + Veh) was also included (without platelets). The live T cell count significantly declined in the T cells + P + cAb group in comparison with the T cells + Veh group, showing the suppressive effect of platelets on T cells (Fig. 7 D). However, in the anti–TLT-1 treated platelet coculture set, the live T cells were comparable with the vehicle control group (Fig. 7 D). The significantly higher T cell numbers in the presence of anti–TLT-1 treated platelets were confirmed to be the result of the increased CD8 T cell fractions (Fig. 7 E). The CD4 T cell fractions did not change in TLT-1 antibody group (Fig. 7 F) as compared with Veh (cAb). This further demonstrates the importance of platelet-derived TLT-1 in NSCLC patients. This provides direct evidence that platelet TLT-1 induces CD8 T cell death, and antibody-mediated targeting of TLT-1 can rescue patient CD8 T cells. CD8 T cells play a key role in restricting tumor growth (Bertrand et al., 2015; Eyles et al., 2010; Spranger et al., 2013). These results support our other in vivo findings and re-emphasize the potential of TLT-1 as an antitumor therapeutic target. Our data not only demonstrate that NSCLC platelet TLT-1 suppresses CD8 T cells but also provides direct evidence that extracellular TLT-1 can be targeted using an antibody-based therapeutic approach to prevent platelet-mediated suppression of patient CD8 T cells in NSCLC.

## Discussion

In the present study, our findings demonstrate that TLT-1, a platelet-expressed protein, can interact with T cells, and its elevation in circulation can suppress CD8 T cells promoting tumor progression. The platelets from NSCLC patients show a distinct activation phenotype, expressing and releasing TLT-1. This phenotype is also observed in mice xenografted with tumors derived from NSCLC patients. The syngeneic mouse model study revealed that the TLT-1 elevation in circulation is able to suppress the effector CD8 T cells and promote tumor progression. The antibody-mediated targeting of TLT-1 reduced tumor growth and enhanced the tumor infiltration of CD8 T cells in mice, while blocking platelet TLT-1 in NSCLC patient samples ex vivo could rescue the CD8 T cells. Mechanistically, we have also identified CD3ε as the receptor for TLT-1 on T cells. Finally, we also demonstrate the prognostic significance of TLT-1 measurement and its association with NSCLC patient survival.

Recent studies have demonstrated the importance of normal functioning “systemic” antitumor immunity driven by T cells (Kamphorst et al., 2017; Poggio et al., 2019). Immunotherapies that target immune checkpoints (such as PDL1/PD1) aimed at reverting tumor-related immunosuppression are emerging as a standard of care for advanced NSCLC and other cancer types (Egen et al., 2020; Grosser et al., 2019; Sharma and Allison, 2015). We adopted a reverse translational approach to explore the role of platelets in tumor-associated immunosuppression, initially studying the platelets from NSCLC patients, followed by more mechanistic in vivo (mouse) and in vitro experiments. Previous studies have suggested that platelets may play a role in tumor growth and metastasis (Borsig et al., 2001; Coupland et al., 2012; Haemmerle et al., 2017; Palumbo et al., 2005; Xu et al., 2018), with one suggesting that platelets can suppress T cells via TGFβ–GARP axis (Rachidi et al., 2017). However, most of these studies have focused on mouse models.

Our platelet investigations revealed distinct platelet phenotypic alterations in NSCLC patients characterized by partial activation, leading to increased TLT-1 release. We also demonstrate that these patient platelets display a distinct resistance to apoptosis along with enhanced mitochondrial integrity, which is opposite to what is observed in other diseases such as diabetes (Jain et al., 2022). Previously, in ovarian cancer patients, platelets were found to have improved mitochondrial health as compared with healthy donor platelets (Wang et al., 2015). Like tumor-associated stroma, blood platelets may also be “transformed” in cancer. This implies that tumor-induced platelet transformation may not be limited to NSCLC. Tumor-associated phenotypic transformation of cells is often accompanied by transcriptome alterations (Riedel et al., 2016). Platelets from cancer patients including NSCLC have been previously reported to show a considerably altered transcriptome profile and can carry tumor signatures, possibly educated through extracellular tumor exosomes (Best et al., 2015; Nilsson et al., 2011), which can explain the tumor-associated platelet phenotypic changes observed by us. Importantly, the partial activation phenotype, including the upregulation and increased release of TLT-1 from NSCLC patient platelets, may have roles beyond hemostasis and thrombosis. The increase in soluble TLT-1 in NSCLC further encouraged us to explore the interactions of TLT-1 with other circulating immune cells.

As demonstrated by our syngeneic mice tumor model, extracellular TLT-1 can interact with T cells in vivo. Circulating T cells in the blood have been reported to be important in antitumor response, with levels of circulating T cells correlating positively with enhanced patient survival and tumor infiltration (Kamphorst et al., 2017; Lee et al., 1999). Moreover, circulating cells would be one of the first to be exposed to the platelet-released TLT-1. Our mouse tumor studies provide evidence in support of TLT-1 suppressing CD8 T cells, resulting in tumor progression. Conversely, in our study groups, we did not find significant alteration of CD4 T cells in response to TLT-1 administration. In our human in vitro assays, TLT-1 inhibition was able to revert the platelet-induced CD8 T cell suppression. Interestingly, a large-scale computational analysis of tumor-associated CD8 T cell dysfunction also listed TLT-1 as one of the markers associated with CD8 T cell dysfunction in a diverse range of cancers (Jiang et al., 2018), which further supports our findings. Our mice and human studies consistently displayed the suppressive effect of TLT-1 on CD8 but not on CD4 T cells. Thus, while both CD4 and CD8 T cells are important in antitumor response, we found that CD8 T cells are much more responsive to TLT-1 elevation and its inhibition as compared with CD4 T cells, which can resist the suppressive effect of TLT-1 such as that observed on CD8 T cells. In view of the complexity and functional diversity of CD4 T cell subtypes—Th1, Th2, Th17, and regulatory T cells—future studies could elucidate the precise response of each CD4 cell subtype to TLT-1. Our assays show that the activation of CD4 T cells in vivo is not affected by TLT-1 elevation (mouse T cell stimulation assay). Therefore, the role of CD4 T cells appears to be very limited in mediating the protumor effects of TLT-1 as compared with that of CD8 T cells. As the tumor microenvironment is complex, other factors such as macrophage density and angiogenesis also play important roles in tumor progression. We did not find any differences in vascular density or macrophages in mice tumors in response to TLT-1 elevation in vivo. This further emphasizes that the tumor-promoting effects of elevated soluble TLT-1 are primarily mediated by T cells.

Platelet MHC-I molecules have been shown to suppress immune thrombocytopenia (Aslam et al., 2008; Gouttefangeas et al., 2000; Guo et al., 2014). Recently, a role for platelet MHC-1 in suppressing CD8 T cells in sepsis has also been reported (Guo et al., 2021; Marcoux et al., 2021). This suggests that the platelet–T cell interactions are complex. Though the role of platelet MHC-I in antitumor immunity remains to be explored, its therapeutic targeting may not be feasible based on the essential role of MHC-1 for a normal functioning immune system.

Our findings provide evidence for TLT-1 acting as a soluble immunocheckpoint ligand for CD8 T cells. TLT-1 bears a conserved IgV domain, which is also found in immunocheckpoint ligands belonging to the B7 family (CD80/86, PDL1; Collins et al., 2005; Gattis et al., 2006; Kundapura and Ramagopal, 2019). The B7 family soluble proteins indeed use IgV domain for interacting with inhibitory receptors such as CTLA-4 and PD-1 on T cells and have been recognized to cause CD8 T cell suppression (Basak et al., 2020; Collins et al., 2005). Through our mechanistic study, we identified T cell surface CD3ε to be the receptor for platelet-released TLT-1 likely responsible for a suppressive effect. Further, we observed that TLT-1 was able to induce AICD-like phenotype in CD8 T cells. Previously, studies on OKT3 antibody (binds to CD3ε) have demonstrated the induction of apoptosis in activated T cells or AICD (Shi et al., 1990), further supporting our observations. T cell apoptosis is one of the major underlying mechanisms behind compromised antitumor immunity and consequent tumor progression (Dong et al., 2002; Weisshaar et al., 2022). NF-κB pathway and its responsive genes have been implicated in CD3ε-induced AICD (Huang et al., 2018). While results from our gene expression arrays demonstrate the involvement of the NF-κB pathway, further investigations are needed to elucidate the role of NF-κB signaling and other downstream events in response to TLT-1 in T cells. CD3ε, an important part of the TCR complex, is also known to play a role in thymic development and other developmental phases of immunity (Bettini et al., 2017; DeJarnette et al., 1998). As our study demonstrates the interaction of TLT-1 with T cells in cancer, its role in normal immune development as well as other pathologies will not be surprising. Beyond its role in antitumor immunity, TLT-1 is involved in other platelet functions as well. Soluble TLT-1 has been previously shown to bind to fibrinogen and facilitate platelet aggregation (Washington et al., 2009), and thrombotic events are a known complication in NSCLC patients (Deschenes-Simard et al., 2021; Sheng et al., 2013). While our present study aims to explore the role of TLT-1 in tumor immunity, it would also be interesting and important to dissect the role of TLT-1 in coagulation in cancer patients, as TLT-1 inhibition could be also potentially relevant in the management of thrombosis risk in cancer patients.

The association of increased platelet TLT-1 levels with reduced survival of NSCLC patients, both in our cohort and in previous datasets, supports its potential as a therapeutic target. The therapeutic potential of targeting TLT-1 was demonstrated with an antibody that targets the IgV domain (N-terminal) of TLT-1. The significant reduction in mice tumor size in anti–TLT-1 antibody group further emphasizes the clinical translational potential of targeting TLT-1 in cancer. The decrease in soluble TLT-1 levels in antibody-administered mice suggests that the elevation of soluble circulating TLT-1 may be a key factor in mediating its effects on T cells. The increased CD8 T cell infiltration in tumors of antibody-administered mice further supports our findings. The tumor microenvironment is highly complex, and more detailed investigations would provide more insights. Taken together, these results suggest that elevated platelet TLT-1 plays a key role in suppressing the antitumor response of CD8 T cells, thereby promoting tumor progression.

Our study provides critical novel insights into cancer-associated immunosuppression, but it also has some limitations. While we demonstrate that NSCLS patients display a distinct phenotype, more extensive studies can identify the tumor mediator(s) responsible for triggering the NSCLC platelet transformation. Further, diverse larger cohort studies would help in delineating the role of tumor type, tumor stage, and individual factors (genetics and environment) leading to the upregulation of TLT-1 in platelets.

In summary, our study identifies a platelet TLT-1–mediated immunoregulatory mechanism that drives T cell suppression. The underlying mechanism appears to be through direct interaction between platelet-derived TLT-1 and CD3ε present on the T cell surface. Our finding that high TLT-1 expression negatively correlates with NSCLC patient survival supports its clinical significance. The effectiveness of antibody-based inhibition of the extracellular TLT-1 makes it an attractive antitumor therapeutic target in NSCLC, and possibly other solid tumors. Our results also pave the way for investigating the role of TLT-1 in other pathological conditions, e.g., autoimmunity, transplant rejection, and hematological malignancies, wherein TLT-1 administration could be tested in conditions that require immunosuppression.

## Materials and methods

### Study approval

All human studies were approved by the Yale Human Investigation Committee (#1005006865). Informed consent was obtained from each subject and conformed to the principles set out in the World Medical Association Declaration of Helsinki and the Belmont Report. These are requirements for the Yale Human Investigation Committee. All mouse procedures were approved under Institutional Animal Care and Use Committee (IACUC) at Yale, and all guidelines were duly followed (IACUC #2017-11413).

### Human subjects

Patients (age range 52–75 yr, *n* = 42) with NSCLC undergoing treatment at the Smilow Cancer Hospital/Yale Cancer Center were recruited for the platelet studies. All patients were NSCLC stage IVA or advanced. Detailed patient characteristics are provided in Table S1. The study was approved by the Yale Institutional Review Board and performed in compliance with all relevant ethical regulations, and all patients consented in writing. Age-matched healthy control volunteers have also consented. Demographic details are provided in Table S2.

### Isolation of human platelets

Platelet isolation requires many precautions, which is due to their sensitivity to postisolation procedures, as discussed by us recently (Tyagi et al., 2022). Venous blood (20 ml) was drawn from patients or healthy age-matched volunteers in EDTA tubes (BD Biosciences). Platelet-rich plasma (PRP) was prepared from freshly drawn blood by centrifugation at 250 *g* for 15 min. The top three-fourths PRP was transferred to a fresh tube and spun again at 250 *g* for 5 min to settle contaminating RBC/WBCs, and the supernatant was transferred to a fresh tube. The platelets were pelleted by centrifuging at 700 *g* for 8 min, and the pellet was gently resuspended in calcium-free platelet washing buffer (103 mmol/liter NaCl, 5 mmol/liter KCl, 1 mmol/liter MgCl_2_, 5 mmol/liter glucose, 36 mmol/liter citric acid, 0.35% BSA, pH 6.5) or in PBS depending on the assay. Platelet counts were measured manually and were diluted with the same washing buffer if needed. These washed platelets were then used for multiple assays.

### FC for platelet surface expressed proteins

Platelet surface expression of proteins was analyzed using the direct staining method (fluorophore-conjugated primary antibodies) as described previously (Schmitz et al., 1998). Briefly, the washed platelets were diluted (1:5) with 1% BSA and were incubated with saturating concentrations of fluorophore-conjugated test antibodies for 40 min at room temperature. The incubation was terminated by dilution and the samples were acquired immediately on an LSRII flow cytometer (BD Biosciences) at standard settings. The originally unconjugated antibodies (anti–TLT-1) as well as their respective isotype control antibodies were conjugated using fluorophore conjugating kits (Abcam) as per the manufacturer’s instructions. The platelets were gated based on forward and side scatter plots on a logarithmic scale, and purity was confirmed by gating on CD41 positive events. A total of 30,000–50,000 events were acquired at “low” flow rate for each assay tube and were kept constant for every set. The data were analyzed by FlowJo software v10.1, and median fluorescence intensities for each set were recorded.

### Transmission electron microscopy (EM)

Platelet ultrastructure was studied as described previously (Jain et al., 2022). Freshly isolated washed platelets were fixed with 2.5% glutaraldehyde in 0.1 M cacodylate buffer (pH 7.2) containing 2% sucrose followed by washing and embedding in Epon. The ultrathin sections were stained with uranyl acetate and lead citrate and examined under FEI Tecnai Biotwin (LaB6) transmission electron microscope at Yale’s EM facility.

### PMV assay

FC-based platelet release of microvesicles was studied as described previously (Mitsios et al., 2006). The FSC threshold was adjusted to view smaller-sized particles (excluding the smallest-sized noise) in logarithmic plots. Standard-sized fluorescent beads (Spherotech) and FSC threshold settings were used to differentiate microvesicles from noise.

### Platelet mitochondrial integrity assay

Platelet mitochondrial damage was tested by nonyl-acridine orange (NAO) dye–based method as described previously (Ferlini and Scambia, 2007). The NAO dye binds specifically to mitochondrial cardiolipin if its structure is intact. In early apoptotic cells, the cardiolipin gets oxidized (loss of mitochondrial structural integrity; NAO is no longer able to bind), which ultimately leads to mitochondrial membrane depolarization and apoptosis (Mileykovskaya et al., 2001). NAO negative platelets thus include early as well as late apoptotic cells. NAO (2.5 µM) is added to platelets in PBS and incubated for 20 min at 37°C followed by dilution (1:4 in PBS) and spun at 700 *g* for 7 min. The pellet was resuspended in 1% BSA (in PBS) solution and immediately studied (30,000–50,000 events/sample) on an LSRII (BD) on the FITC channel.

### Western blotting

Platelet Western blotting was performed as previously described (Jain et al., 2022). Washed platelets were lysed in radioimmunoprecipitation assay buffer with the addition of protease and phosphatase inhibitors (Sigma-Aldrich). Western blot analysis was performed using 20 μg protein to evaluate the expression of apoptosis-associated proteins. The cell lysates were subjected to 10% SDS–polyacrylamide gel electrophoresis. The protein was electrophoretically transferred to nitrocellulose membrane and then blocked with 5% milk in Tris-buffered saline solution containing 0.5% Tween-20. The membranes were probed using the respective antibodies, incubated with horseradish peroxidase–conjugated anti-rabbit IgG, anti-goat IgG, or anti-mouse IgG. Antibody against TLT-1 was obtained from R&D Systems. Active caspase-3 was purchased from Abcam. Antibodies against Bcl-2 and Bax were purchased from Cell Signaling Technology. Bands were viewed using the enzyme-linked chemiluminescence detection reagents. GAPDH or tubulin were used as loading controls.

### Platelet surface activation assay

For human platelets, the PAC1 assay was used to analyze the activation of αIIbβ_3_ surface receptor on platelets. The PAC1 antibody binds specifically to the activated conformation of human αIIbβ_3_ receptor and not to its resting form (Shattil et al., 1985). The FITC-conjugated PAC1 antibody (BD Biosciences) was added to washed platelets as per the manufacturer’s instructions, and after 30 min of incubation at room temperature, the reaction was stopped and the events were acquired on an LSRII.

### ELISA

Human plasma was isolated by centrifuging freshly drawn blood at 2,000 *g* for 10 min and frozen below −80°C until further analysis. The ELISA kit (R&D Systems) was used for measuring TLT-1 concentration as per the manufacturer’s instructions. The microparticles were isolated from thawed plasma samples. The microparticle pellet was dissolved in Tris-saline buffer and used for ELISA. The microparticle-depleted supernatant fraction was also used for comparison.

### Platelet confocal microscopy

The assay was performed as previously described (Jain et al., 2019). Briefly, washed platelets were allowed to adhere and get activated/spread on a round glass-bottomed dish at 37°C for 1 h. After fixation, washing, blocking, and staining steps, the FITC-conjugated antibody for CD41 was used to visualize platelets along with PE-conjugated anti–TLT-1 in the dark. After washing excess antibodies, the stained platelets were imaged by Nikon Eclipse Ti confocal microscope using 100× oil immersion lens. The images were captured and analyzed using Volocity v6.3 software.

### RNA extraction and RT-PCR

The platelet lysates for RNA isolation were prepared using TRIzol reagent (Invitrogen). RNA extraction was performed using Zymo Direct-zol kit (with DNase treatment). The purity of each platelet RNA preparation was assessed by PCR analysis of platelet (presence of GPIIb) and leukocyte (absence of CD45) markers, as described previously (Landry et al., 2009). RT-PCR was used to measure mRNA expression levels of TLT-1. RNA isolated from platelets was reverse-transcribed using a cDNA synthesis kit (Promega). PCR was performed with 5× supermix (Invitrogen) and specific primers (purchased from IDT) using a Bio-Rad thermocycler as per the manufacturer’s instructions. Expression levels were calculated as fold change overexpression of the housekeeping gene (β-tubulin) for each sample.

The primers used were as follows: TLT-1 Fw 5′-GAG​GAA​GAA​GAA​GAG​ACC​CAT​AAG-3′ and Rev 5′-GCC​CAG​TGT​GTA​ATG​GTA​GAA-3′.

### Generation of primary xenografts

Discarded NSCLC tumor tissues from patients (*n* = 3) undergoing therapeutic surgical procedures were obtained fresh and transferred to the laboratory on ice. The samples were deidentified and were used as per approved protocols from the Institutional review board. Under aseptic conditions, the tumor samples were processed as described previously (Daniel et al., 2009). The immunocompromised (Rag2^−/−^, IL2r^−/−^) MISTRG-6 (humanized for cytokines M-CSF, IL3, SirpAlpha, Tpo, and IL6) mice (Rongvaux et al., 2014; Yu et al., 2017) were housed at the Yale Animal Facility under defined guidelines on a regular diet. The live tumor cells (viable counts in range 2–12 × 10^5^/ml) were counted and injected subcutaneously on the right flank of the mice (*n* = 6), while the control group received buffer only. The tumor growth was monitored and measured by a digital caliper for 4 wk and then the mice were sacrificed after blood collection.

### Mice subcutaneous B16F10 tumor model

C57BL/6 mice (aged 4–6 mo) were housed at the Yale Animal Facility on a standard diet. All animal protocols were approved by Yale’s IACUC. Both male and female mice were included in the study. The mouse firefly luciferase gene expressing tumor cell line (B16F10-Luc) was purchased (Imanis Life Sciences) and cultured as per the manufacturer’s instructions. These bioluminescent syngeneic cells (3 × 10^5^/100 μl per mouse) were injected subcutaneously in immunocompetent mice as detailed previously (Overwijk and Restifo, 2001). The tumor presence was confirmed after 3 d under the animal bioimaging system (PerkinElmer), and any mouse with unusually small or large tumors (due to possible variations during injections) was not included in the treatment groups. Mice with similar-sized tumors were randomly grouped into two sets. One set (+TLT-1) received purified active recombinant mouse TLT-1 (Creative Biomart; 55 μl resuspended in PBS) by tail vein once a week, starting at day 7 after tumor, while the other set received the recombinant mouse IgG in same volume of PBS simultaneously. The once-a-week dose was selected based on our observation that recTLT-1 after a single in vivo dose in mice became undetectable after 10 d. The tumor size was regularly measured by a digital caliper and tumor volume was calculated by the standard formula *V* = (*W*^2^ × *L*)/2, where *W* is the width and *L* is length of the tumor measured by the caliper. Tumor growth was also monitored by bioluminescent imaging. The mice were sacrificed after 4–5 wk depending upon tumor size. The tumor, spleen, and lung tissues were immediately harvested. The images of the whole lungs were captured. The lung and tumor tissues were fixed in cold paraformaldehyde (4%) while spleens were weighed and transferred to culture media.

### Isolation of mouse platelets

Blood (0.1 ml) was collected from retro-orbital plexus of mice using the capillary method into sodium citrate (10% vol/vol). The blood was diluted and spun at 200 *g* for 8 min at room temperature to obtain PRP. The PRP was again centrifuged at 100 *g* for 5 min to settle the contaminating WBCs. The purity was checked manually under the microscope using a hemocytometer and was further confirmed by FC. The PRP was appropriately diluted with Tyrode’s Hepes buffer (calcium free) and used for further assays.

### Mouse plasma isolation and ELISA

The mouse plasma was isolated from whole blood as described previously (Jain et al., 2019). The ELISA for soluble TLT-1 (R&D Systems) was performed as per the manufacturer’s instructions.

### FC-based mouse blood T cell analysis

Mouse whole blood (0.1 ml) collected by capillary from retro-orbital plexus was mixed with sodium citrate and processed for RBC lysis using a mild RBC lysis buffer (BioLegend) as per the manufacturer’s instructions. The pelleted cells were resuspended in 10× volume of cell surface staining buffer (BioLegend) and divided into assay tubes for surface staining antibodies for TCRb, CD4, and CD8 (all primary conjugated). For intracellular staining, a cell permeabilization/fixation buffer was used to resuspend cells followed by staining with antibodies for TCRb, CD8, and IFNγ along with isotype control set. The mice from all comparison groups were included on each day of performing the assay to avoid any day-to-day variations. The samples were immediately acquired on LSRII (BD), and data were analyzed by FlowJo v10.

### Mouse tissue immunofluorescence

The excised tumors were fixed in 4% paraformaldehyde overnight and incubated with 30% sucrose (in PBS) solution for 48 h. Tumors were transferred to optimum cutting temperature media and frozen on dry ice. The cryosections of excised mouse B16F10 tumors from the primary site were processed (washing, blocking) for immunofluorescence staining by anti-TCRb, anti-CD8 (BioLegend), anti-luciferase (Abcam), and anti-IFNγ antibodies, which was followed by incubation with secondary antibodies (AlexaFluor 488 for TCRb, AlexaFluor 647 for luciferase, AlexaFluor 405 for CD8, and AlexaFluor 568 for IFNγ). The stained sections were viewed under a spinning disk confocal microscope (Nikon Eclipse Ti) and the images were captured using Volocity v6.3. Immunofluorescence quantitations were performed using Volocity software by independent viewers blinded to the study groups.

### Mouse splenic T cell culture and analysis

Single-cell suspension of splenocytes were produced by removing red blood cells with ACK lysis buffer. Cells were counted and stained directly with conjugated antibodies for T cell markers (CD4-Pacific Blue, CD8-AlexaFluor 488, CD62L-PE, CD44-AlexaFluor 647) in the presence of FcBlock (BD Biosciences). We also stimulated 2 × 10^6^ splenocytes/ml with soluble anti-CD3 and anti-CD28 antibodies (0.5 µg/ml ea.) in the presence of 1× brefeldin A (BioLegend) for 6–12 h prior to fixable viability staining with eFluor-780 (Thermo Fisher Scientific) and surface staining with CD4-Pacific Blue and CD8-AlexaFluor 488. After extensive washes and treatment with fixation and permeabilization buffer (Thermo Fisher Scientific), we stained cells with PE-conjugated anti-mouse IFNγ. A minimum of 50,000 cells were acquired satisfying live cell forward and side scatter parameters using an LSR II flow cytometer and the data were analyzed with FlowJo software. The threshold for cytokine-positive T cells was manually set using similarly gated T cells incubated without stimulation.

### TLT-1 antibody administration in vivo in subcutaneous tumor mice

The anti–TLT-1 IgG2b antibody (R&D Systems), mouse specific for N-terminal IgV domain, was administered at a preoptimized dose of 0.45 mg/kg body weight in 100 μl sterile PBS via the intraperitoneal route. The injections were given at a 5-d interval starting on day 7 after the subcutaneous tumor injections. Isotype control IgG2b antibody (R&D Systems) was also used in the control groups at a dose of 0.45 mg/kg body weight. If any mice rejected the tumors naturally after 6 d or had unusually increased growth, those were excluded from the study. The tumors were monitored and measured every 2 d and after 5 wk of tumor growth using in vivo tumor imaging.

### Isolation of human T cells

The freshly drawn blood from subjects in EDTA vacutainers was processed for peripheral blood mononuclear cell isolation using Ficoll-Paque reagent (GE Healthcare). The isolation of total human T cells or CD4 and CD8 T cells was carried out using negative selection magnetic bead-based kits as per the manufacturer’s instructions (StemCell Technologies). The purity of isolated cells was confirmed by FC assays.

### T cell RNA isolation and quantitative PCR (qPCR) array

The T cells after culture were spun down and lysed using TRIzol reagent and stored at −80°C. The qPCR array kit for human inflammation and cancer genes (Qiagen) was purchased and used as per the manufacturer’s instructions. The RNA isolation was performed followed by the cDNA synthesis step. The qPCR was performed on Bio-Rad thermal cycler. The obtained dCt values were used for array data analysis as per the manufacturer’s instructions. A set of five housekeeping genes (*ACTB*, *B2M*, *GAPDH*, *HPRT1*, and *RPLP0*) were used to calculate the dCt values for the expression of studied genes. A heat map was generated with the mean of fold regulation values of samples.

### Co-IP assay

The Jurkat cell line (ATCC) was used for Co-IP study as these are the human T cell lines. The cells were cultured in RPMI-1640 complete medium in 10% FBS at 37°C and 5% CO_2_ and harvested after reaching 80–85% confluency. The intact cells were incubated for 15 min with Fc-tagged (at C-terminal) purified TLT-1 or with IgG as control, and the lysate was prepared in mild IP lysis buffer (not boiled) as per Co-IP Dynabead based kit protocol (Thermo Fisher Scientific), followed by incubation of total 1 mg protein with beads and wash steps as per manufacturer’s instructions. Immunoblotting was performed for individual targets along with the non-IP lysate controls (inputs).

### Coculture of human platelets and T cells

Platelets and T cells were freshly isolated from NSCLC patients (as described). The purity of both cell types was confirmed by FC. The autologous platelets and T cells were counted, and T cells were cultured with (1:100) or without platelets in RPMI-1640 complete medium in 10% FBS at 37°C and 5% CO_2_ for 24–48 h. The anti–TLT-1 antibody (R&D Systems) or isotype control IgG (R&D Systems) was added (5 µg/ml) to platelet suspension before initiating coculture. The CD4 or CD8 T cells were washed and identified by primary conjugated antibodies in the FC assay.

### Cancer data mining

The computational evaluation of the clinical potential of TLT-1 expression in cancer in terms of its effect on patient survival (OS and FP) using previously uploaded datasets was performed using a webtool—kmplotter (Lanczky et al., 2016). This webtool also provides the beeswarm curve for the gene of interest to show the used expression threshold and option to exclude biased arrays from the analysis. The analysis of NSCLC patient data (for previous cohort studies) was performed for both adenocarcinoma and squamous types. The data mining for TLT-1 (*TREML1*) expression, T cell infiltration, and immunocheckpoints expression in tumor tissue was performed using the TIMER webtool as described previously (Li et al., 2017).

### Human lung tissue immunofluorescence and dual IHC staining

The cryosections of lung biopsy samples from NSCLC patients were prepared by standard OCT-based method. The sections from tumor-rich or tumor-poor areas of the same patient were processed for immunofluorescence staining using anti-CD3 (Biolegend), anti-CD8 (Abcam), anti-panCK (Abcam), and anti–TLT-1 (R&D Systems) primary antibodies followed by immunofluorophore-tagged secondary antibodies (Alexa Fluor 488 for CD3 primary, Alexa Fluor 594 for TLT-1 primary, BV-421 for CD8 primary, and Cy-5 for panCK primary). The stained sections were viewed under a spinning disk confocal microscope (Nikon Eclipse Ti) under the same software settings for all sections to be compared and images were captured using Volocity v6.3.

The dual immunohistochemistry (IHC) was performed by using kit-based method for dual staining as per the manufacturer’s instructions (Vector Biosciences). The two primary antibody combinations i.e., CD4 and TLT-1 or CD8 and TLT-1 were used followed by conjugated secondary antibodies and counterstaining using Gill’s hematoxylin stain. The tumor sections were observed under a Nikon 80i microscope. The tumor nests were used for quantification by the observer blinded to experimental groups in consultation with a pathologist.

### Statistical analysis

Values are presented as mean ± SEM. Comparisons between two study groups were performed using the nonparametric two-tailed Mann–Whitney test for non-normally distributed data. Student’s *t* test was used for normally distributed data. Data sets with three or more groups were analyzed Kruskal–Wallis test followed by Dunn’s multiple comparisons test (non-parametric) or one-way ANOVA with Tukey multiple comparisons test. For correlation, Pearson’s test was used to determine significance and the trend line was determined by nonlinear regression. The patient survival analysis was performed using logrank-based Mantel–Cox test. Analysis was performed with Prism 8.0 software (GraphPad Software, Inc.).

### Online supplemental material

Fig. S1 shows platelet purity, gating strategy, TLT-1 mRNA, protein quantitation, and PAC1 control in NSCLC platelets along with platelet PMV FC plot, apoptosis FC plots, quantitation and apoptosis pathway Western blots, and quantitation of PMV versus soluble TLT-1 Elisa. Fig. S2 shows similarity in recTLT-1 and native platelet TLT-1 and binding and effect of recTLT-1 on mice T cells, along with gating strategy. Fig. S2 also shows mouse platelet TLT-1 in mice with B16 tumor, in vivo T cell changes, and the gating strategy for cultured mice splenocytes. Fig. S3 shows mouse plasma soluble TLT-1 quantitation in B16 tumor model and CD4 and CD8 TILs in mouse tumors in response to in vivo antibody administration. Fig. S4 shows the association of TLT-1 expression with reduced survival in NSCLC and patient subgroups along with dataset patient group characteristics, H&E sections, and dual IHC section images along with quantitation. Fig S5 shows the results of TLT-1 Co-IP for undetected receptors. Table S1 shows the demographic and clinical characteristics of NSCLC study patients. Table S2 shows demographic characteristics of healthy donors.

## Supplementary Material

Table S1 shows demographic and clinical characteristics of NSCLC study patients.

Table S2 shows demographic and clinical characteristics of healthy control volunteers.

SourceData F1 contains original blots for Fig. 1.

SourceData F6 contains original blots for Fig. 6.

SourceData FS1 contains original blots for Fig. S1.

SourceData FS2 contains original blots for Fig. S2.

SourceData FS5 contains original blots for Fig. S5.
