# Supplementary material for: Platelet-derived TLT-1 promotes tumor progression by suppressing CD8+ T cells
Source: J Exp Med. 2022 Oct 28;220(1):e20212218. doi: 10.1084/jem.20212218 (PMC9814191; doi:10.1084/jem.20212218)
Supplement: Table S1 — shows demographic and clinical characteristics of NSCLC study patients. [file JEM_20212218_TableS1.docx]

**Table S1.** The demographic and clinical characteristics of NSCLC study patients

| Characteristic | | |  | Number of patients |
| --- | --- | --- | --- | --- |
| Total (n) | |  |  | 42 (100%) |
| Age (median ± SD) | | 63.1 ± 6.6 |  |  |
| Sex | | Female |  | 30 (71%) |
|  | | Male |  | 12 (29%) |
| Smoking | | Current/former |  | 34 (81%) |
| Thrombosis | | DVT or SVT |  | 3 (7%) |
|  | |  |  |  |
|  | |  |  |  |
| Stage | | IV |  | 42 (100%) |
| Distant metastasis | | |  | 31 (74%) |
| Histology | | |  |  |
|  | Adenocarcinoma | |  | 34 (81%) |
|  | Squamous | |  | 2 (5%) |
|  | unknown | |  | 6 (14%) |
| Molecular status (mutation) | | | |  |
|  | KRAS | |  | 6 (14%) |
|  | EGFR | |  | 5 (12%) |
|  | ALK-rearrangement | | | 2 (5%) |
|  |  | |  |  |
