# Supplementary material for: Platelet-derived TLT-1 promotes tumor progression by suppressing CD8+ T cells
Source: J Exp Med. 2022 Oct 28;220(1):e20212218. doi: 10.1084/jem.20212218 (PMC9814191; doi:10.1084/jem.20212218)
Supplement: Table S2 — shows demographic and clinical characteristics of healthy control volunteers. [file JEM_20212218_TableS2.docx]

**Table S2.** The demographic and clinical characteristics of healthy control volunteers

| Characteristic | | |  | | Healthy control volunteers | |
| --- | --- | --- | --- | --- | --- | --- |
| Total (n) | |  | |  | | 25 (100%) |
| Age (median ± SD) | | 60.1 ± 8.1 | |  | |  |
| Sex | | Female | |  | | 14 (56%) |
|  | | Male | |  | | 11 (44%) |
| BMI (kg/m^2^ , mean ± SD) | |  | |  | | 26.72 ± 5 |
| Smoking | | Current/former | |  | | 7 (29%) |
| Comorbidities | |  | |  | |  |
| *Diabetes* | |  | |  | | 2 (8%) |
| *Hypertension* | |  | |  | | 3 (12%) |
| *Coronary artery disease* | |  | |  | | 1 (4%) |
| *History of malignancy* | |  | |  | | 0 (0%) |
| *History of thrombosis* | |  | |  | | 1 (4%) |
|  |  | |  | |  | |
