# Supplementary figures and images for: Platelet-derived TLT-1 promotes tumor progression by suppressing CD8+ T cells
Source: J Exp Med. 2022 Oct 28;220(1):e20212218. doi: 10.1084/jem.20212218 (PMC9814191; doi:10.1084/jem.20212218)

Source Data Main **Figure 1**

Main **Fig 1B**

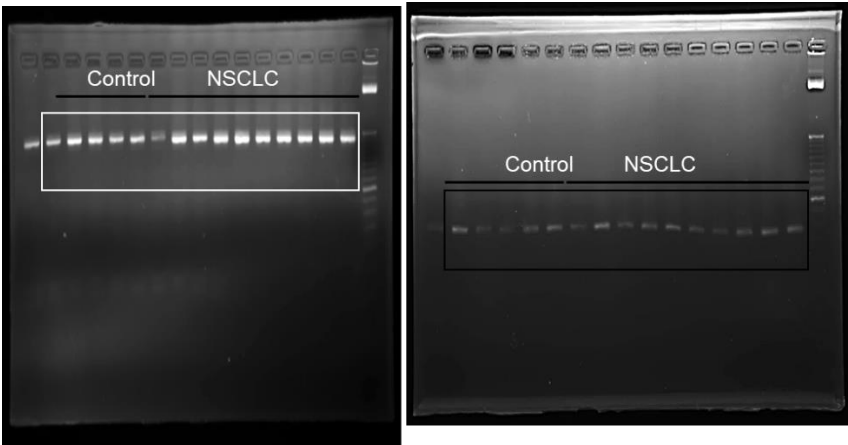

**Fig 1C**

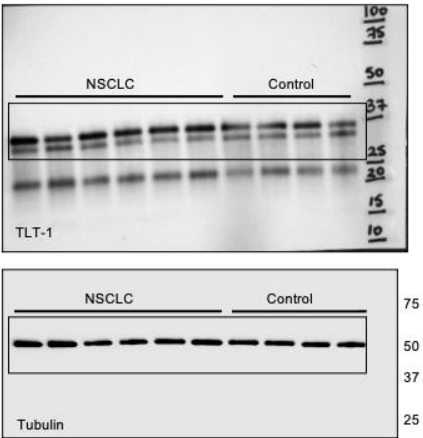

Supplement: SourceData F1 — contains original blots for Fig. 1. [file JEM_20212218_SourceDataF1.pdf]

Source Data Figure 6

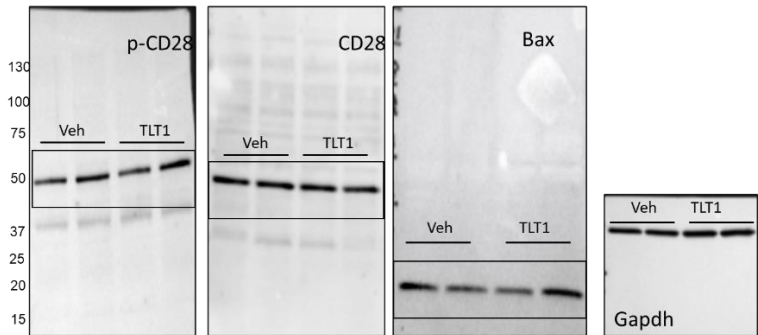

Main Fig 6C

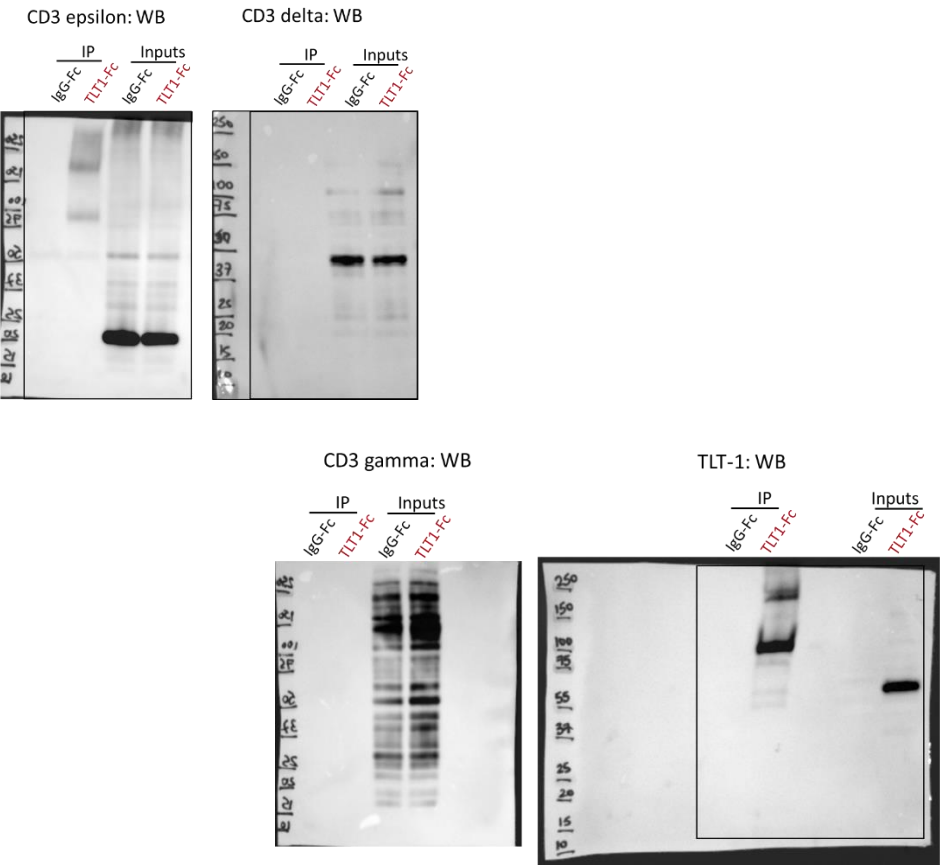

Supplement: SourceData F6 — contains original blots for Fig. 6. [file JEM_20212218_SourceDataF6.pdf]

SourceData **Fig S1**

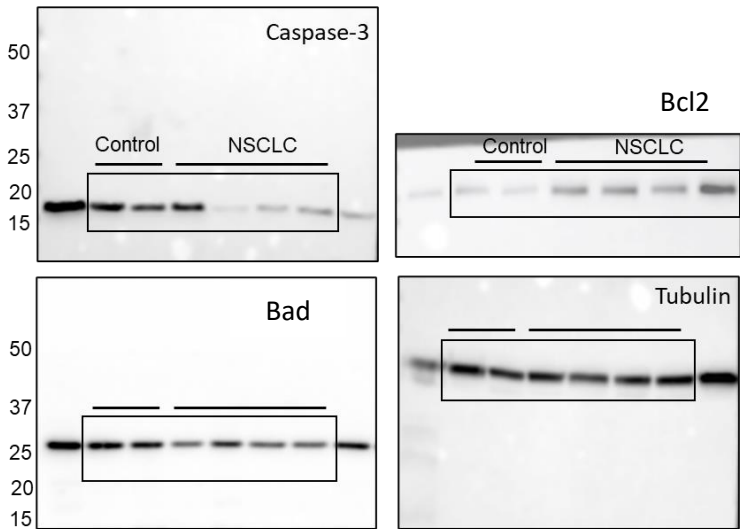

Supplement: SourceData FS1 — contains original blots for Fig. S1. [file JEM_20212218_SourceDataFS1.pdf]

## Suppl **Fig S2**

Fig S2A Mouse TLT-1 WB

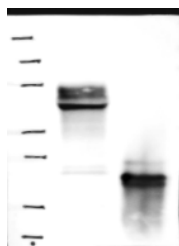

Supplement: SourceData FS2 — contains original blots for Fig. S2. [file JEM_20212218_SourceDataFS2.pdf]

Suppl Fig S5

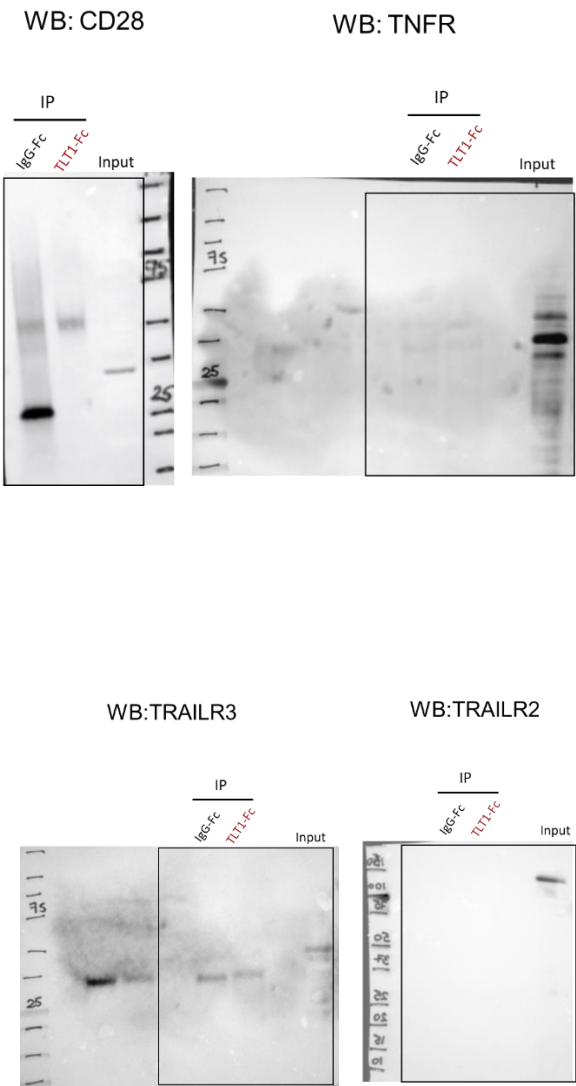

Supplement: SourceData FS5 — contains original blots for Fig. S5. [file JEM_20212218_SourceDataFS5.pdf]
